# Improving route development using convergent retrosynthesis planning

**DOI:** 10.1186/s13321-025-00953-1

**Published:** 2025-02-27

**Authors:** Paula Torren-Peraire, Jonas Verhoeven, Dorota Herman, Hugo Ceulemans, Igor V. Tetko, Jörg K. Wegner

**Affiliations:** 1 In-Silico Discovery, Research & Development, Johnson & Johnson, Beerse, 2340 Belgium; 2 Institute of Structural Biology, Molecular Targets and Therapeutics Center, Helmholtz Munich - Deutsches Forschungszentrum Für Gesundheit Und Umwelt (GmbH), Neuherberg, 86764 Germany; 3 In-Silico Discovery, Research & Development, Johnson & Johnson, Cambridge, 02142 US

**Keywords:** Computer-aided synthesis planning, Convergent routes, Retrosynthesis prediction

## Abstract

Retrosynthesis consists of recursively breaking down a target molecule to produce a synthesis route composed of readily accessible building blocks. In recent years, computer-aided synthesis planning methods have allowed a greater exploration of potential synthesis routes, combining state-of-the-art machine-learning methods with chemical knowledge. However, these methods are generally developed to produce individual routes from a singular product to a set of proposed building blocks and are not designed to leverage potential shared paths between targets. These methods do not necessarily encompass real-world use cases in medicinal chemistry, where one seeks to synthesize sets of target compounds in a library mode, looking for maximal convergence into a shared retrosynthetic path going via advanced key intermediate compounds. Using a graph-based processing pipeline, we explore Johnson & Johnson Electronic Laboratory Notebooks (J&J ELN) and publicly available datasets to identify complex routes with multiple target molecules sharing common intermediates, producing convergent synthesis routes. We find that over 70% of all reactions are involved in convergent synthesis, covering over 80% of all projects in the case of J&J ELN data.

**Scientific contribution**

We introduce a novel planning approach to develop convergent synthesis routes, which can search multiple products and intermediates simultaneously guided by state-of-the-art machine learning single-step retrosynthesis models, enhancing the overall efficiency and practical applicability of retrosynthetic planning. We evaluate the multi-step synthesis planning approach using the extracted convergent routes and observe that solvability is generally high across those routes, being able to identify a convergent route for over 80% of the test routes and showing an individual compound solvability of over 90%. We find that by using a convergent search approach, we can synthesize almost 30% more compounds simultaneously for J&J ELN as compared to using an individual search, while providing an increased use of common intermediates.

**Supplementary Information:**

The online version contains supplementary material available at 10.1186/s13321-025-00953-1.

## Introduction

Compound synthesis is a crucial and time-consuming part of drug discovery, starting from the validation of hit compounds coming out of a target screening exercise, towards the design of structurally related molecules to explore the structure-activity relationship (SAR) target space. This process involves the identification of a suitable synthesis path typically through a process known as retrosynthesis. Retrosynthesis involves hypothetically breaking down a compound via disconnections into progressive reactants until a set of purchasable or easily synthesizable compounds is reached [1]. Multiple approaches have been developed to address retrosynthesis from a machine-learning perspective [2–5], which aims to aid experts in the task of selecting a sequence of chemical reactions that can be applied to a target compound to arrive at commercially available starting materials.

Within computational retrosynthesis approaches, there are two main aspects, the single-step model, which indicates which reaction is most relevant to a molecule, and the multi-step synthesis planning algorithm, which guides the search to establish the combination and order of the reactions [6]. For the latter, different approaches have emerged to address the expansive search, generally following a heuristic to guide the search with methods such as proof-number search [7, 8], Monte-Carlo Tree Search [9–12], which relies on a combination of exploration and exploitation to explore the search, A* search [13, 14] which takes a global view of the task aiming towards synthesizable molecules, and self-play approaches [15, 16] which train a learned policy through multiple simulated experiences.

However, there is an important caveat with these synthesis planning approaches as medicinal chemists typically work in libraries of compounds, where multiple compounds are designed and synthesized simultaneously to explore the activity space of a target of interest [17–19]. This library synthesis is not reflected in common multi-step approaches, which generally focus on the synthesis of a singular compound rather than enabling the design of convergent routes via common intermediates.

A few previous approaches have explored the mutual synthesis of compounds of interest, commonly as a post-hoc analysis step. Recently, Fromer et al. [20] proposed an approach using ASKCOS [21] to prioritize compounds suggested for synthesis based on their potential for batch synthesis along with cost. Pasquini et al. [22] developed a method to combine precalculated retrosynthesis routes for multiple compounds from varying sources. Gao et al. [23] approached the issue as a mixed integer problem using retrosynthetic routes from ASKCOS, going on to select synthesis routes for all WHO essential medicines while minimizing the number of reactants and reagents required [24]. Molga et al. [25] explored the extension of Chematica [26] to multiple target compounds within the search, with the ability to produce a single route for multiple compounds or select the most readily synthesizable compounds for a batch of target compounds. Lastly, Xie et al. [14] extended the Retro*[13] approach towards a graph-based solution, instead of tree-based, to allow the inference of singular retrosynthetic routes for multiple compounds, introducing an additional GNN to avoid circular paths.

In this work, we address this problem by proposing a graph-based multi-step approach to identify retrosynthetic routes for multiple compounds simultaneously, producing convergent routes. This approach prioritizes routes applicable to all target molecules where possible while also suggesting routes for those compounds that cannot be convergently synthesized. Moreover, to ensure the chemical feasibility of our approach, we develop a dataset of convergent routes based on the USPTO dataset [27] and Johnson & Johnson Electronic Laboratory Notebook (J&J ELN) datasets. By additionally implementing batch inference, we can produce convergent retrosynthesis routes for up to hundreds of molecules, identifying a singular convergent route for multiple compounds in the majority of compound sets.

## Methods

In this section, we discuss the two core methodologies developed in this work. Firstly, the curation of the convergent routes dataset establishes a collection of retrosynthetic routes based on reaction data. This details the synthesis of multiple target molecules, under a shared retrosynthetic route using common reactant molecules across the route. Secondly, we focus on the multi-step synthesis planning framework, which discusses convergent retrosynthetic routes to allow the joint synthesis of a library of compounds. The routes are developed using the scores from a single-step model, while biasing towards compounds shared across multiple target molecules to encourage convergence across the retrosynthetic routes.

### Convergent routes dataset

Convergent routes are synthesis routes comprised of multiple target molecules resulting from common intermediates. We develop a pipeline to identify and extract these convergent routes from reaction data. Starting from the reaction data, products and reactants are identified based on atom-mapping. Using the atom-mapped reactions, we split any compound on the reactant side into reactants and reagents depending on their contribution to the product. Any compound on the reactant side which forms at least 20% of the product is considered a reactant. All remaining compounds are assumed to be reagents and discarded. The reaction data is then split based on document identifiers so that reactions carried out together are considered a joint document. Importantly, the reaction data is not deduplicated at this stage, given that the same reaction can occur across multiple documents.

For each document, we create a directed graph where the molecules are represented as nodes (*V*) and reactions between molecules are represented as edges (*E*), however we defer from adding additional reaction nodes as in previous works [28, 29] instead focusing solely on molecule nodes. The graph is set up from a retrosynthetic standpoint where the children of a node are the reactants required for the synthesis of the parent node. Each reaction from a document is added to the graph by adding the molecules individually as nodes and connecting nodes with the relevant edges for the reaction, such that a retrosynthetic reaction with a single product and two reactants will become a parent node with two outgoing edges to each reactant. Once all reactions are added to the directed graph, the graph is then traversed to identify weakly connected components, these are subgraphs where all the nodes are connected through some path, irrespective of the direction of the edges, each extracted subgraph is treated as an individual synthesis graph.

The target molecules and building blocks of each synthesis graph can then be identified. Given a node, $v_i$, if $v_i$ has no incoming edges $(\delta ^-(v_i)=0)$ it will be considered a target molecule since the molecule has not been developed further. If $v_i$ has no outgoing edges $(\delta ^+(v_i)=0)$ the node will be considered a building block since the molecule has no prior reactions. If $v_i$ has multiple incoming edges $(\delta ^- ( v_i) >1)$, from multiple target molecules, then it is considered a common intermediate, this definition works in conjunction to that of a building block, such that a building block may also be considered a common intermediate. Given the focus on convergent routes, all synthesis graphs that do not contain common intermediates are discarded so that all remaining synthesis graphs are convergent routes.

Two main concerns when using reaction data are the ambivalence of reaction direction and the multiple approaches to synthesizing a single molecule. Concerning reaction direction, there can be instances where the same reactant and product combination can be synthesized in both directions $\{(v_i,v_j ),(v_j,v_i )\}$. In this case, if possible, we discard the least common reaction direction, if this is not possible due to lack of data we discard the synthesis graph given that the reaction direction cannot be resolved. Additionally, in reaction data, there are cases in which a single compound was synthesized more than once through different reaction pathways, leading to a cycle within the synthesis graph. In this case, the synthesis graph is discarded since the more optimal reaction path cannot easily be established, ensuring all synthesis graphs are directed acyclic graphs (DAGs). Lastly, once the cleaned convergent synthesis graphs have been established, we ensure that the target molecules within each graph are not simply stereoisomers and that there are no duplicated graphs across the convergent routes dataset.

We refer to the convergent routes extracted from the reaction data as the experimentally validated routes throughout the text. We refrain from referring to these as ground truth or other terms are given that multiple retrosynthetic paths can be used to successfully synthesize a target molecule or a compound library, and so the extracted routes can only be considered an example of a chemically successful synthesis path.

### Multi-Step Synthesis Planning

**Fig. 1:**
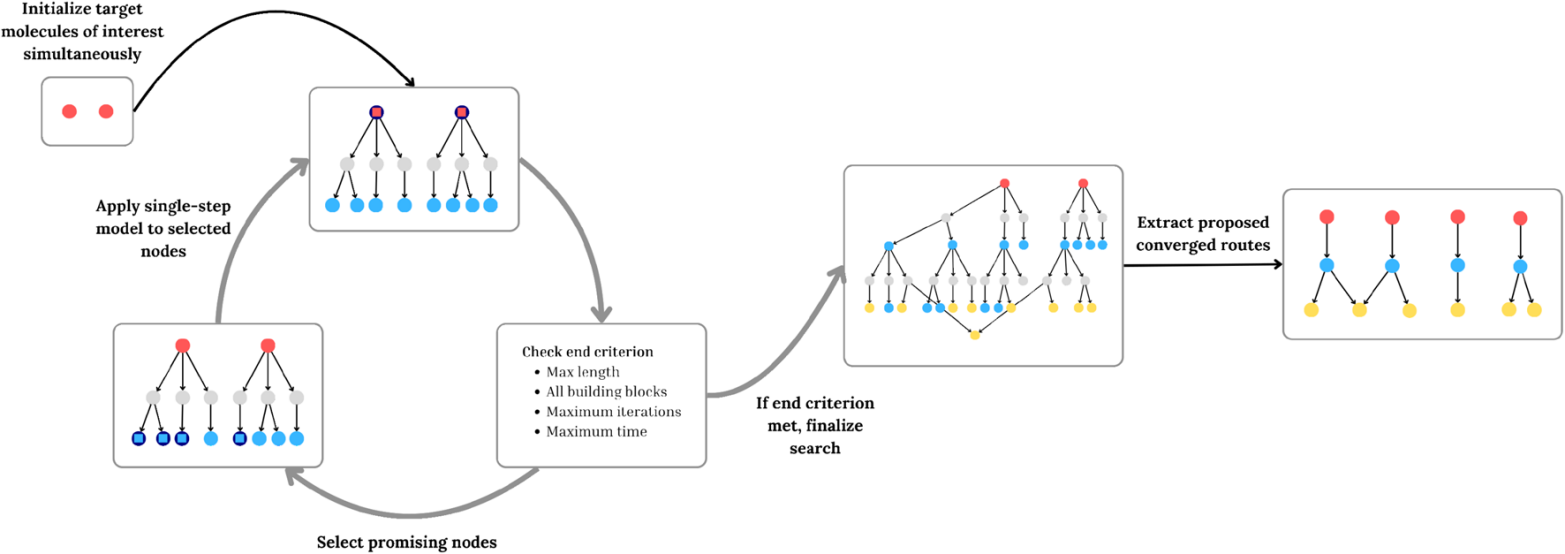
Multistep search process. All target molecules (red) are initiated simultaneously. At the first iteration, K sets of reactants are proposed for each target molecule, for each set of proposed reactants a reaction node (grey) is added followed by the relevant reactants (blue). The n most promising molecule nodes (circled in dark blue) are followed up, and K sets of reactants are proposed for each, with the same process continued iteratively until all end nodes are building blocks (yellow) or the time or iteration limit is reached. The retrosynthetic routes are then extracted from the search graph. The hypothetical example shown consists of two target molecules, with three sets of reactants proposed for each molecule node (K) and four molecule nodes (n) followed up with a maximum of two iterations. Only a sample of the potential extracted routes is shown

The multi-step search is based on a directed graph, exploiting this format for simultaneously instantiating multiple target molecules. The multi-step search contains two types of nodes, molecule nodes and reaction nodes. The search is guided by a single-step model that proposes reactants given a product. When starting a retrosynthetic search, all target molecules are instantiated simultaneously as molecule nodes (Fig. 1), differing from using a dummy node to connect target molecules [23, 25]. At the first iteration, all target molecules are considered promising nodes, and K sets of reactants are proposed for each target molecule. For each target molecule ($m_t$), K child reaction nodes are created ($\delta ^+ (m_t) = K$). Each reaction node (*c*) will have an associated set of reactants, where the proposed reactant set is a collection of one or more required reactants ($r = {r_1,r_2,...,r_{j-1},r_j}$). From each reaction node, a molecule node (*m*) is added for every molecule in the proposed reactant set, ($\delta ^+ (c) = |r|$), such that every molecule node will have a maximum of K outgoing edges and every reaction node will have the same number of outgoing edges as the number of molecules in the proposed reactant set. If a molecule node already exists in the search, the reaction node will be linked to the existing molecule node. If two reaction nodes originating from the same molecule node lead to the same set of proposed reactants, then only one instance of the reaction node and consequent molecule nodes will be added to the search. If any of the proposed reactants for a reaction node are considered invalid, no molecule nodes will be added to the reaction node.

The scores of the proposed reactants are used to select the top n most promising nodes at each iteration. Promising nodes are selected based on the product of the single step model probabilities along the linear path between each target molecule and a given end node. Where a linear path $P(v_i,v_j)$ is defined as the consecutive edges linking two nodes,

1 $$\begin{aligned} P(v_i, v_j) = \{v_i, ..., v_j\} : v_iv_{i+1} \in E \end{aligned}$$

we calculate the linear path between an end node $m_i$, where $m_i$ is a molecule node (*M*) and not a building block (*B*), has no outgoing edges and the shortest distance to any target molecule is below the predefined maximum depth (*L*),

2 $$\begin{aligned} m_i = {\left\{ \begin{array}{ll} v_i \in M \\ Out_i = 0 \\ v_i \notin B \\ min(|P(m_{t_i}, m_i)|) < L \\ \end{array}\right. } \end{aligned}$$

and a target molecule $m_{t_i}$. The relevant target molecules are defined as molecules that form part of the original target molecule set (*T*) and have a path between the target molecule and the end node in question ($m_i$).

3 $$\begin{aligned} m_t = {\left\{ \begin{array}{ll} v_i \in T \\ \exists P(v_i, m_i) \\ \end{array}\right. } \end{aligned}$$

Considering all paths between $m_{t_i}$ and $m_i$, we calculate the product of the reaction nodes along each path,

4 $$\begin{aligned} x = {\left\{ \begin{array}{ll} v_i \notin M \\ v_i \in P(m_{t_i}, m_i) \\ \end{array}\right. } \end{aligned}$$

keeping the maximum score of these for each $m_{t_i}$ and average across all $m_t$.

5 $$\begin{aligned} score_{m_i} = \frac{\sum _{j=1}^{|m_t|} max(\prod _{k=1}^{|P(m_{t_j}, m_i)|} \mathbb {P}(x_k))}{|m_t|} \end{aligned}$$

By calculating the score for each relevant end node ($score_{m_i}$) we can rank those and select the top n highest scores as promising nodes to be followed up by the single-step model. This is also described as pseudocode in Supp Figs. 8, 9. We focus on using a scoring-based expansion, skewing towards selecting reactants which are shared across multiple target molecules. The multi-step search is implemented such that the single-step model can use the native GPU inference setting, allowing for faster batch inference of the top n most promising nodes.

The same process is carried out until any stop criteria are reached. These include maximum time or iterations per molecule as well as all potential molecule nodes being explored and flagged as either building blocks (*B*) or the maximum route length (*L*). Both maximum time and iterations are set according to the number of target molecules in the compound library, due to the use of batch inference the total number of iterations is further divided by the batch size.

Once the search is finalized, the proposed routes must be extracted. The search graph is pruned to remove any paths that do not end in building blocks, leaving only routes with actionable proposals, as routes that do not end in building blocks cannot be considered solved. The proposed building blocks in the search graph are scored by the product of the probabilities of the single-step model at each reaction step from every target molecule to a given building block, averaged across all target molecules. The building blocks with the highest score are explored first. We parse the search graph to extract the relevant route by identifying the highest-scoring linear route from each unexplored molecule to an end node. The process continues until a complete synthesis tree is established. This is carried out until all potential building blocks are explored or the maximum time limit is reached. The proposed routes are ranked using the product of the single-step model probabilities of each reaction step within the route. The routes are then summarized by removing the reaction nodes, as such the final routes contain exclusively molecule nodes and directed edges linking together the molecule nodes.

Of note, in this work we focus exclusively on using the scores from the single-step model, skewing the scores towards proposed reactants that are present across multiple target compounds. We differ from using other search algorithm implementations such as MCTS or Retro* [9, 12, 13] given their further computational complexity in establishing retrosynthetic routes. Previous work [28, 30, 31] has shown that MCTS requires a higher number of model calls while providing a similar solvability of compounds, whereas Retro*, when using an additional learned scored has minimal effect on the solvability, while requiring further training data [13, 30]. Here, we propose an initial work based on solely the single-step model scores, however the framework is set up such that an additional learned score or different single-step model could be included.

### Experimental Details

To assess the multi-step planning approach, we use the extracted routes from the convergent routes dataset. We create two convergent routes datasets based on J&J ELN [32] data and USPTO [27] data. For the USPTO dataset, we use the full reaction dataset, including both applications and grants subsets. In the case of USPTO, we assume that all data shows positive yield, for J&J ELN data we select only reactions with yield $\ge$ 5% to ensure previously successful retrosynthetic routes. We create a convergent routes dataset for each reaction dataset, we clean and standardize all reactions, stripping salts and canonicalizing using RDKit [33], keeping all reported stereochemistry information. Products and reactants are identified based on the atom-mapping from GraphormerMapper [34]. Project identifiers for J&J ELN and patent identifiers for USPTO are used to delimit documents. From J&J ELN and USPTO, we select 500 and 1000 convergent routes from the respective datasets to create a convergent route hold-out test set.

To train the single-step model, we clean and standardize all reactions, defining products and reactants as with the convergent routes dataset. From there, we remove all reactions that form part of routes in the respective convergent routes test set, then deduplicate the remaining routes, removing any reactions with multiple products. We carry out a random 80%/10%/10% train/validation/test split with each reaction dataset. We fine-tune the pre-trained Chemformer [2] model based on each reaction dataset, the Chemformer model is fine-tuned to propose reactant SMILES based on product SMILES. We use the default hyperparameters, and training methodology provided by the original publication and detailed in their GitHub repository.

For the multi-step search, we consider the target molecules from each convergent route a library of molecules, giving one compound library per convergent route. In the case of the individual compounds comparison, we run each molecule individually, irrespective of the convergent route of origin. Unless otherwise stated, the building block set is composed of all end nodes across each route from the respective convergent test set. It comprises almost 5000 molecules for J&J ELN and 10,239 molecules for USPTO convergent test sets. We explore ten molecule nodes of interest (*n*) at each iteration and set the beam size (*K*) of the single-step model to 5, using the beam sampling of the single-step model to calculate the scores of each node as defined in the previous section. Only five potential reaction steps for each node are proposed since accurate next reaction steps are commonly found within the top 5 suggestions of the single-step model [35]. For the search, we set a maximum of 2 min, maximum route length (depth) of eight reaction steps and 300 iterations per molecule i.e. rounds of selecting and following up on promising nodes. Additionally, we set a limit of 300 target molecules per convergent search. All multi-step searches are run on a single Tesla T4 GPU with 8 CPU nodes. All multi-step searches are run on a single Tesla T4 GPU with 8 CPU nodes.

### Evaluation

The proposed routes are analyzed using solvability, accuracy, and F1 score as metrics (Table 1). These routes consist exclusively of molecule nodes and edges linking these together, which can be interpreted as reactions. We address two types of solvability, complete and partial. Complete solvability refers to whether the top-N route is a singular to convergent route that jointly synthesizes all target molecules within a search. Partial solvability, on the other hand, refers to whether all target molecules feature in at least one route up to top-N, irrespective of whether the compounds are synthesized conjointly. The accuracy metric (Supp Eq. A1) assesses if there is an exact match between the proposed route at top-N and the experimentally validated route from the convergent routes test set. Accuracy requires that the exact compounds are used throughout the route in the same order and is assessed by comparing the reactions (edges) of both routes. We further analyze the intermediate accuracy, which scores whether we identify the same common intermediate in the proposed and experimentally validated route. Accuracy is a very stringent metric, particularly in retrosynthesis, given that a slight change in a molecule, e.g., having a different halogen atom used as a functionality to couple two building blocks, will lead the route to be labeled as inaccurate. We introduce an F1 score to quantify the similarity between the proposed routes and the experimentally validated route, we combine two F1 scores, based on the reactions (edges) and the molecules (nodes). In the case of the reaction F1 score (Supp Eq. A2), true positives are defined as reactions that are correctly identified in the route, false negatives are reactions that are not present in the proposed route compared to the experimentally validated route, and false positives are reactions that are present in the proposed route but not the experimentally validated route. The same concept applies to the molecule F1 score (Supp Eq. A3), except that the target molecules are not included in the comparison to avoid positively skewing the metric towards short routes with multiple target molecules. The F1 score is calculated separately for the reactions (edges) and for the molecules (nodes). The average of these two metrics is used and referred to as the F1 score (Supp Eq. A4) in the text, a visual example of the F1 score calculation is provided in Supp Fig. 10.

**Table 1:** Summary of metrics used to assess retrosynthetic routes

Metric		Description
Solvability	Complete	Top-N route is a singular convergent route that jointly synthesizes all target molecules in compound library
	Partial	All target molecules feature in at least one route up to top-N, irrespective of whether the compounds are synthesized conjointly
	Individual	At least one synthesis route is proposed for a given compound, compounds are addressed individually irrespective of compound library
Accuracy	Route	Exact match between the proposed route at top-N and the experimentally validated route
	Intermediate	Exact match between the proposed common intermediates at top-N and common intermediates in experimentally validated route
	Individual	Exact match between the proposed route at top-N and the experimentally validated route on an individual compound level
F1 score		Averaged harmonic mean of recall and precision based on proposed reactants and reactions as compared to experimentally validated route

To further understand the chemistry of the routes, we additionally assess the reaction names and types of the proposed routes, using NameRxn [36]. We extract the reaction type from each reactants-product pair in the highest-ranked proposed route, carrying out the same process for the experimentally validated route. We compare the set of reaction names from the experimentally validated route with the proposed routes, ignoring instances of Unrecognized reaction type, to assess the reaction type accuracy of the proposed routes. We then map these reaction types to the corresponding reaction class, a higher-level definition, to also calculate the reaction class accuracy.

## Results & discussion

### Convergent routes dataset

**Fig. 2:**
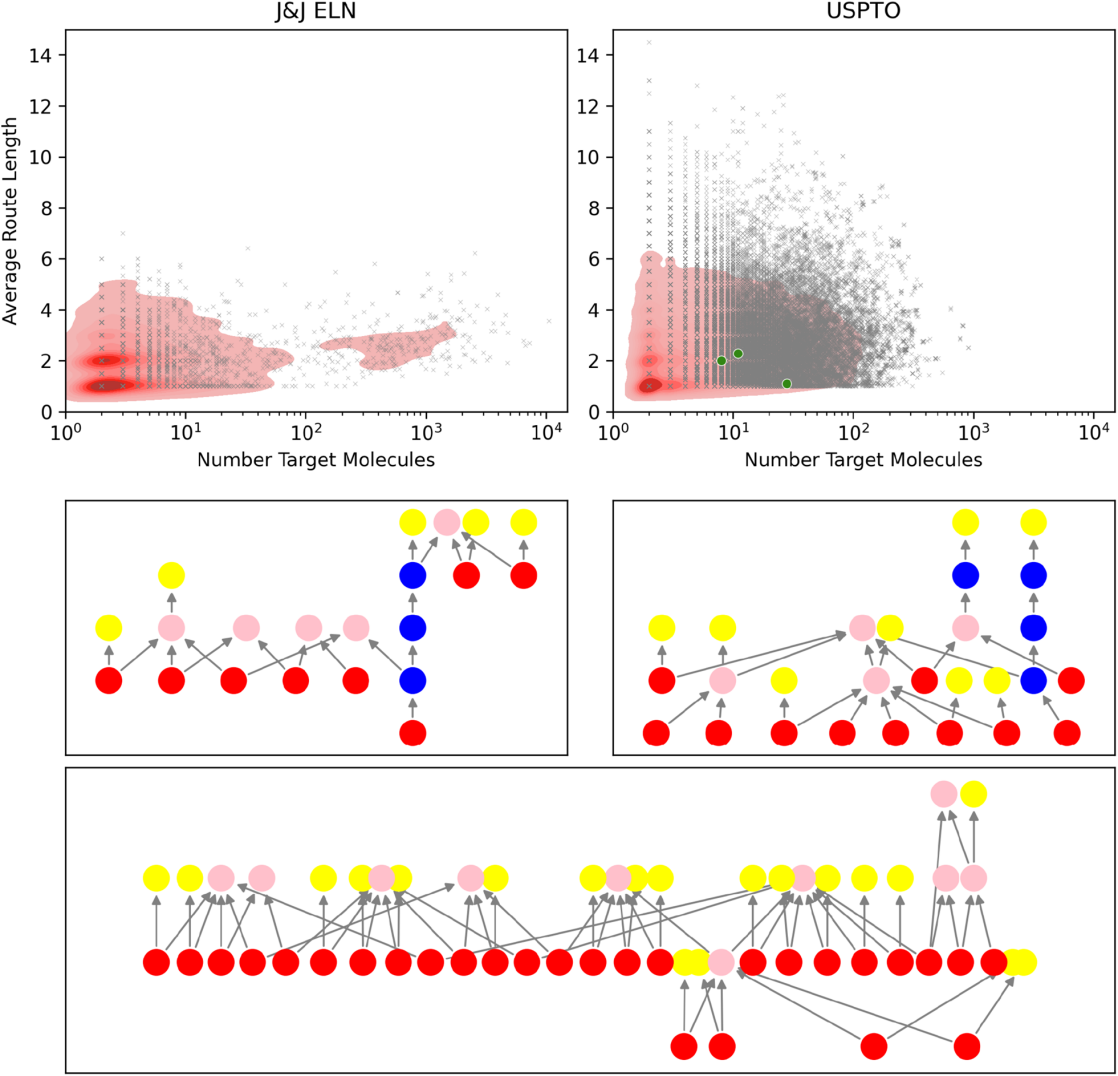
Top panel: Distribution of the number of target molecules and average route length for convergent routes per convergent route from J&J ELN and USPTO. Bottom panels: Example convergent routes from USPTO, red nodes show target molecules, pink nodes are intermediates that are used more than once, blue nodes are intermediates that are only used once, and yellow nodes show building blocks. The shown routes are highlighted in green in the top panel

Using J&J ELN and USPTO data separately we create a convergent route dataset of each reaction dataset. Convergent routes are highly relevant in medicinal chemistry with the majority of syntheses involving multiple target molecules, we find that 79% of all reactions from J&J ELN form part of a convergent route, with 85% of all documents containing at least one convergent route. We identify 94,833 convergent routes within USPTO across all 3.7 million original reactions. In this case, we notice that 70% of all USPTO reactions are involved in convergent routes, with 37% of all documents containing at least one convergent route. This lower document coverage reflects the skewed distribution of the number of reactions per patent within the USPTO dataset, with 27% of all USPTO patents having only one associated reaction and over half of all projects containing four or fewer reactions (Supp Fig. 11), with previous works [28] also retrieving a relatively low number of patents for single molecule retrosynthetic routes. Convergent routes are generally convoluted routes, with respectively 61% and 72% of J&J ELN and USPTO routes having more than two target molecules and more than two reaction steps depth (Fig. 2). Across both J&J ELN and USPTO, most convergent routes have a single common intermediate across all target molecules (Supp Fig. 12). J&J ELN convergent routes tend to have more target molecules, whereas USPTO routes tend to be longer in depth. Importantly, convergent routes are often applied to reduce the number of reactions necessary to synthesize a set of target molecules, thus also reducing the time and cost of the synthesis. In the case of USPTO, we find that the number of reactions required for the synthesis of 988,476 molecules is reduced by 40%, going from 2,883,640 reactions when analyzing the synthesis routes of target molecules individually to 1,770,237 reactions when reusing reactions through a convergent route approach and addressing projects from a compound library perspective. Overall, the high prevalence of convergent routes shows the propensity for synthesis of multiple target molecules instead of focusing on a singular compound of interest.

### Multi-step search

We develop a new multi-step synthesis planning framework that instantiates multiple target molecules simultaneously, with the aim of convergent route development. Using the convergent route datasets developed for J&J ELN and USPTO, we can search for convergent routes for real compound libraries to assess the utility of the approach. We randomly select 500 and 1000 convergent routes from J&J ELN and USPTO, respectively, as the multi-step test set. For the single-step model, we fine-tune the pre-trained Chemformer on each dataset, J&J ELN and USPTO, respectively, using the remaining data to carry out a train/validation/test split.

**Fig. 3:**
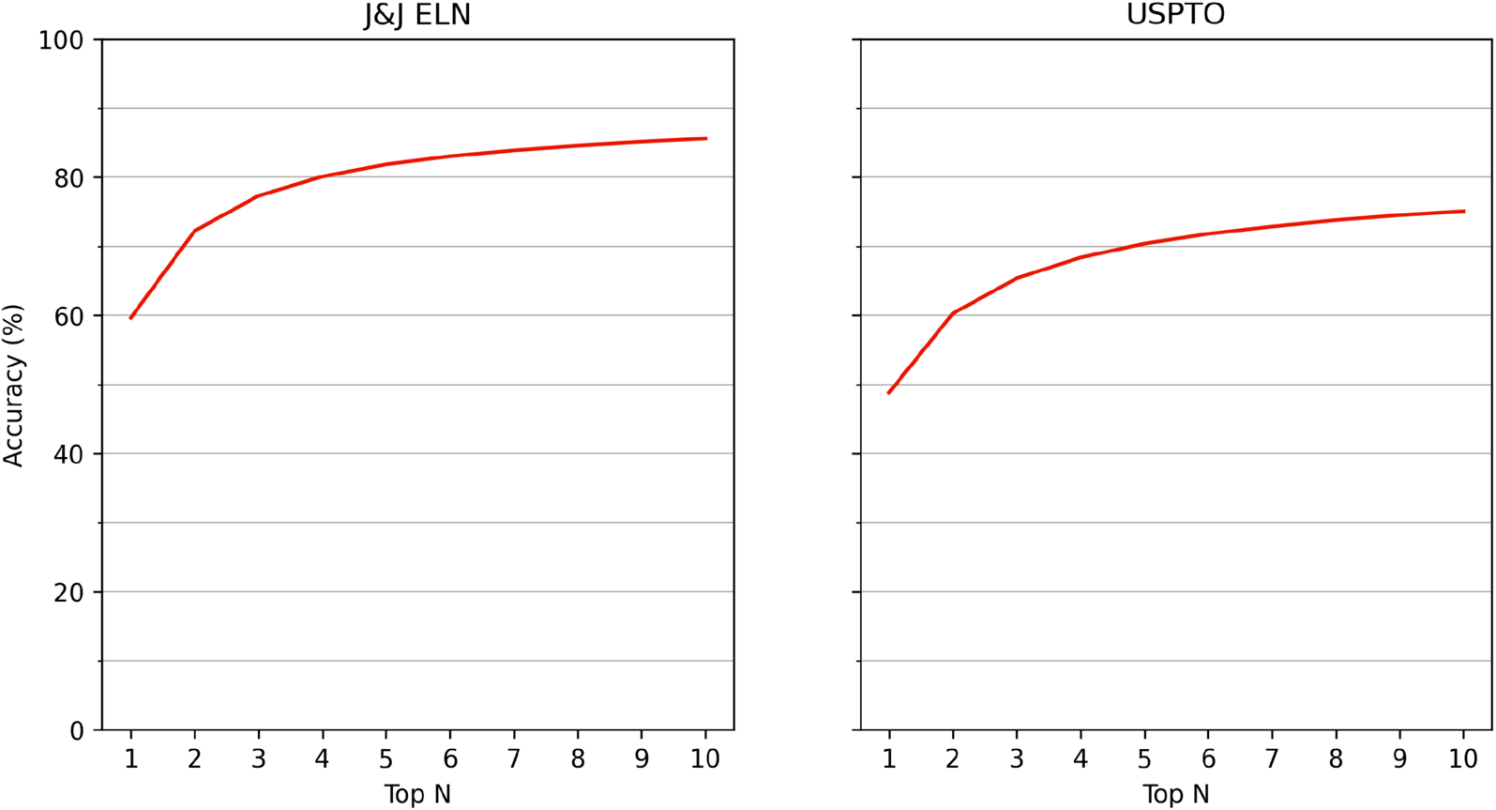
Single-step accuracy of finetuned Chemformer for J&J ELN and USPTO reaction datasets

Both single-step models show a similar pattern of accuracy across the top-N, achieving high accuracy by top-10, particularly in the case of the J&J ELN-trained model. The J&J ELN single-step model reaches 85% accuracy at top-10 whereas USPTO reaches 75% accuracy at top-10, with this pattern of 10% difference present across all top-N (Fig. 3). In the case of USPTO, we see that the model performance is slightly lower as compared to the evaluation on USPTO-PaRoutes [35], which undergoes further data preparation steps. We use these models to guide the respective multi-step synthesis planning for each library of target molecules from the J&J ELN and USPTO test sets.

J&J ELN test set routes tend to be smaller than USPTO convergent routes, given that J&J ELN convergent routes tend to have fewer target molecules and common intermediates (Table 2). USPTO routes tend to have more building blocks, potentially due to using less advanced molecules as starting points. When applying the compound libraries to the multi-step search, the approach proposes 75 and 88 routes per compound library on average for J&J ELN and USPTO, respectively. Interestingly, we have a variety of potential common intermediates identified for each compound library, with 17 unique common intermediate molecule combinations for J&J ELN and 22 unique common intermediate molecule combinations for USPTO within the top-100 routes for each compound library. This shows that the proposed routes show diversity, producing multiple options for the potential synthesis of the compound library. Within the proposed routes, the highest-ranked route tends to have characteristics similar to the experimentally validated route, sharing a comparable number of common intermediates, building blocks, and target molecules per common intermediate (Table 3). Additionally, we see that the proposed routes tend to show a slightly higher number of molecules and reactions while maintaining a similar number of reactants per reaction. This indicates that additional reaction steps are added in the proposed routes, leading to longer synthesis paths overall.

**Table 2:** Average statistics of convergent routes test set

	J&J ELN	USPTO
Target molecules	7.4	9.7
Route Length	2.5	3.3
Common intermediates	2.9	4.0
Building blocks	6.2	10.1
Molecules	16.8	27.3
Reactions	10.6	17.2
Reactants per reaction	1.6	1.6
Target molecules per intermediate	4.3	4.8

**Table 3:** Average statistics of the highest-ranked proposed route

	J&J ELN	USPTO
Target Molecules	5.2	7.8
Route Length	4.2	4.4
Common intermediates	2.3	3.6
Building blocks	6.5	10.3
Molecules	20.7	31.1
Reactions	14.2	20.8
Reactants per reaction	1.5	1.5
Target molecules per intermediate	3.9	4.5

**Fig. 4:**
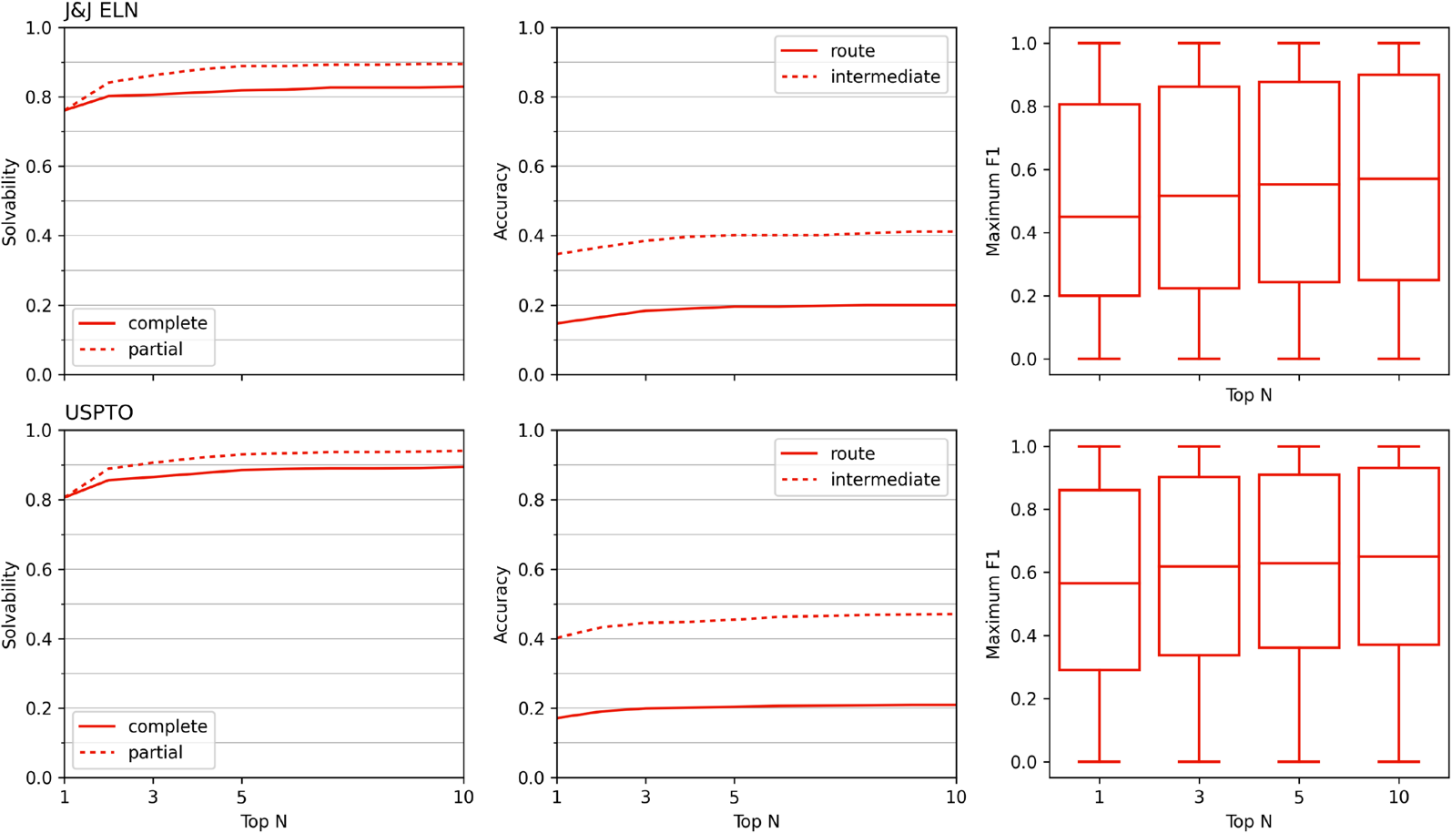
Solvability, accuracy, and F1 score on convergent test set compared to experimentally validated routes

Using these libraries of target molecules as test compounds, the multi-step search can typically identify a route to convergently synthesize all target molecules within each library. This shows that the approach can efficiently produce convergent routes within a single search, leading to more streamlined and cost-effective synthesis. Using the convergent route multi-step search approach, we can produce a single convergent route for 81% of all explored libraries in the case of J&J ELN (Fig. 4) within the top 10 proposed routes, rising to 87.5% when considering solvability for all explored target molecules within each library, irrespective of whether the molecules are synthesized across one or more routes. We find a convergent synthesis route for 89.4% of all USPTO compound libraries, rising to 94.1% for compound libraries where all target molecules are not identified in the same synthesis route. The small difference between complete and partial solvability shows that the approach can suggest convergent routes in the majority of cases, with a lack of synthesizability, i.e., reaching building blocks, being a larger bottleneck than identifying a convergent route across the compound library, with 97.1% and 99% of compounds per compound library forming part of a singular proposed retrosynthetic route for J&J ELN and USPTO.

Importantly, this approach proposes routes for as many target molecules as possible, irrespective of whether they are conjointly synthesized, so that we can maximize the utility of the retrosynthetic routes. When considering the solvability of individual compounds across all compound libraries, we identify retrosynthetic routes for 97.5% and 99.5% of all individual compounds for J&J ELN and USPTO, respectively. This highlights the value of using convergent routes search to increase the solvability of the multi-step search, given that the convergent approach can aid in resolving a greater number of target molecules.

Using the convergent route dataset, we can further explore the accuracy of the proposed retrosynthetic routes. This poses a much more complex challenge, given the numerous alternatives that can be used for compound synthesis [37]. We replicate 20.0% of the experimentally validated routes within the top 10 proposed routes for J&J ELN, with a slightly higher accuracy of 20.9% for USPTO routes (Fig. 3). Interestingly, we correctly identified the common intermediate for 41.1% of J&J ELN and 47.0% of USPTO of the target molecule sets within the top 10 proposed routes. This implies that the suggested routes do not follow the exact reaction steps as the experimentally validated route however they suggest routes similar to those within the experimentally validated routes, particularly considering that we identify the same pattern in both datasets.

**Fig. 5:**
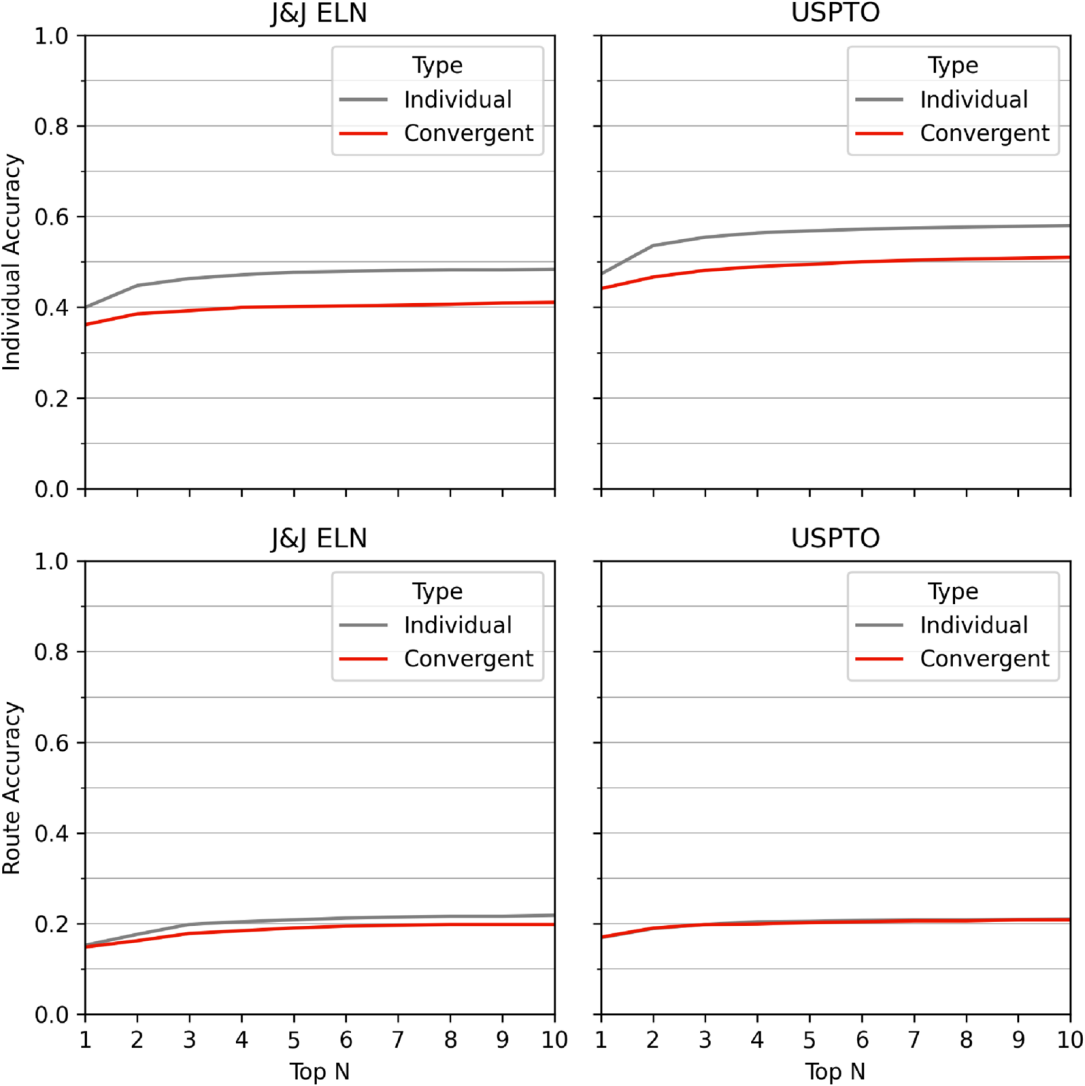
Top: Route accuracy of individual compounds using an individual compound approach and convergent approach, only routes with at least two reaction steps are considered. Bottom: Convergent accuracy based on compound libraries, using an individual approach where if all individual compounds are accurate, then the combined route is assumed to be accurate, and the convergent approach proposes a convergent route

Current works for convergent routes require the combination of precomputed retrosynthetic routes [20, 22]. As such, we can further compare this approach to developing a single route for each compound within the compound library and then combining the routes to identify convergent routes. In this case, the analysis is run individually on each compound within the compound library, where we can also analyze the proposed convergent routes on a per-compound basis. When using the compounds individually we find that we produce routes for 96.4% and 99.2% of compounds individually for J&J ELN and USPTO (Fig. 5), this is slightly below the solvability achieved on a per compound basis for the convergent route approach, which provides 97.5% and 99.5% individual compound solvability respectively.

This indicates that including additional compounds can allow the search to prioritize lower-ranked reactions that it otherwise would not explore within an individual setting. Thus, we find that by using the convergent approach, we can provide routes for a larger proportion of the target molecules overall. However, this does come at a loss for individual accuracy, and we find that running compounds individually, increases individual accuracy by 7% across the board for both J&J ELN and USPTO. Despite the individual compound approach providing a higher accuracy on the individual routes, we noted that when we analyze the accuracy across a compound library, assessing whether all compounds within a compound library accurately replicate the experimentally validated synthesis route, that there is minimal difference in their accuracy. Importantly, the convergent method does not require a combinatorial approach which would incur $N^{m_t}$ route combinations, where N is the number of routes considered per target molecule, and $m_t$ denotes the number of target molecules in the compound library, leading to massive time frames as the number of target molecules increases (Supp Fig. 14). Moreover, we explore the comparison of an additional real world use case where we aim to synthesize the compound libraries using a real-world building block set, eMolecules [38]. Using a random selection of 10% of each compound library test set we produce a complete convergent route for 63% and 74% of J&J ELN and USPTO, respectively. Importantly, when we compare these routes to the alternative of combining the top-1 route from the individual search, under the same testing conditions, we find that through the convergent approach we conjointly synthesize 29% and 22% more compounds from the J&J ELN and USPTO compound libraries while using 20% more target molecules per common intermediate, an increase in the number of target molecules that can be synthesized while reusing the same proposed reactants.

**Fig. 6:**
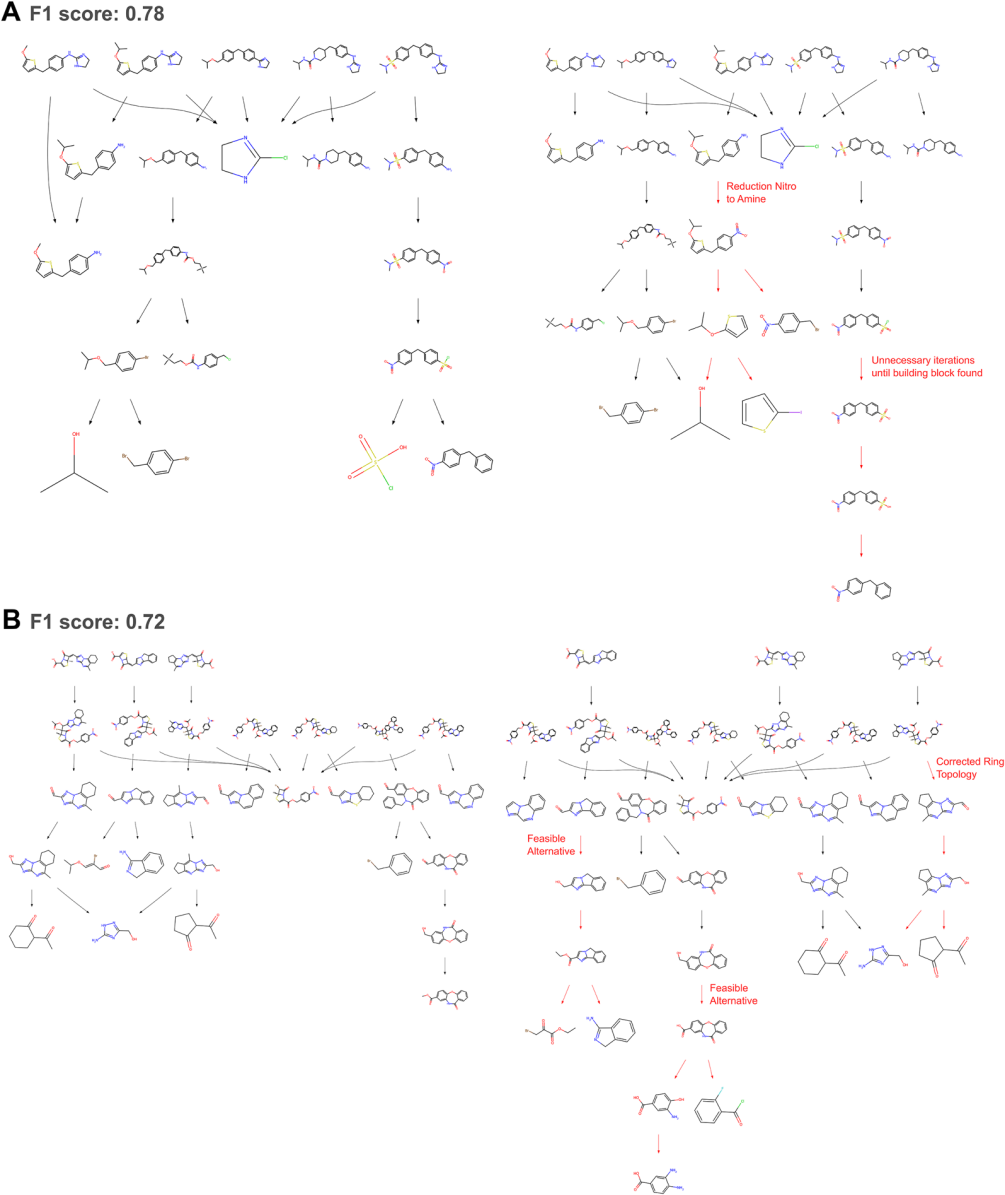
Illustrative examples of experimentally validated retrosynthetic routes (left) and highest scoring proposed route (right) from USPTO, each panel (**A** and** B**) shows a different compound library. Red arrows indicate proposed retrosynthetic steps that differ from the experimentally validated route. Paths described as feasible alternatives were defined as such by a medicinal chemist. Full scale versions of each route are available in Supp Fig. 20 - 23

Solvability and accuracy, however, do not tell the whole story of the suitability of the proposed convergent retrosynthetic routes, given that within chemistry and particularly retrosynthesis [39], multiple paths can lead to the synthesis of a compound. Similarity in routes can be hard to quantify, particularly in the case of convergent routes, given that one change in reactant can lead the entire route to be deemed inaccurate. Here, we propose to use the F1 score as a metric to quantify the similarity of proposed and experimentally validated routes. By calculating and averaging the F1 score of the proposed reactions and molecules, we find that over half of all libraries have an F1 score higher than 0.55 by the top 5 (Fig. 4), and more than 34% of routes within the top 10 have an F1-score over 0.75, in both datasets, the individual molecule and reaction F1 scores can be found in Supp Fig. 15. These routes show minor changes to the experimentally validated route, adding additional reaction steps or proposing an alternative retrosynthetic path for one of the target molecules in the library, as discussed with a medicinal chemist. Figure 6 shows examples of proposed routes which are similar route to the experimentally validated route, showing minor differences in the proposed route. The target molecules use a slightly different retrosynthetic route for early-stage intermediates with greater effects across the convergent route, as noted by the decreasing F1 score.

**Fig. 7:**
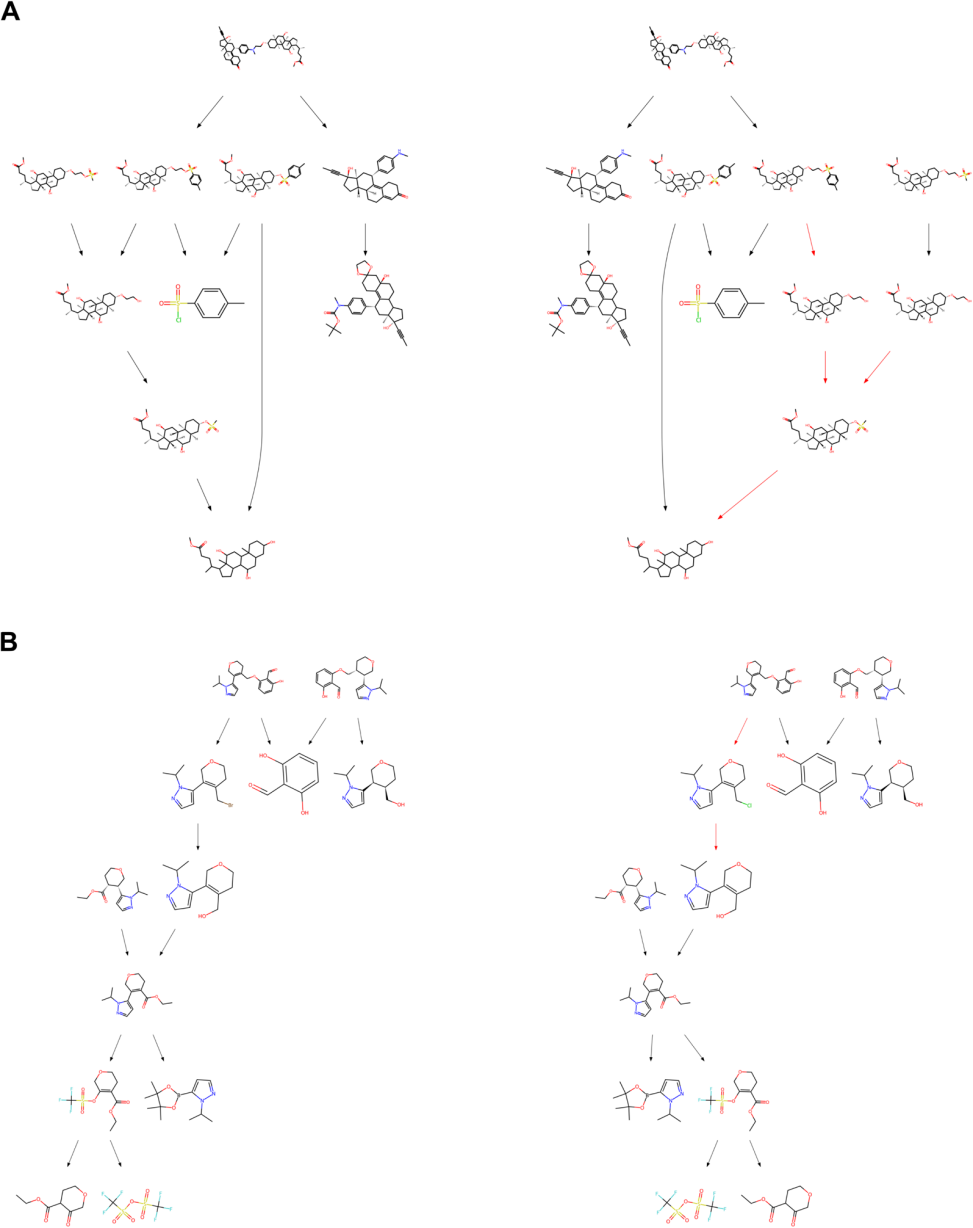
Illustrative examples of experimentally validated route (left) compared to the proposed route (right). In panel** A**, the proposed route differs from the experimental route in two main areas, however, the reaction types and classes matchup between the routes. In panel** B**, the proposed route is deemed inaccurate and has different reaction types but matching reaction classes. Full scale versions of each route are available in Supp Fig. 24–27

Considering the diversity of the convergent routes test set, we can further analyze how routes of varying sizes and characteristics perform. Generally, the number of reaction steps within the convergent route has a more pronounced impact on the F1 score than the number of target molecules (Supp Fig. 16-17), with the F1 score showing a clear downward trend with an increasing number of reaction steps, indicating that the effect of successive reactions has a greater impact than the complexity through the number of target molecules. Additionally, if we consider the similarity between target molecules, as would naturally occur in QSAR studies, we find that more structurally similar compound libraries tend to have a higher F1 score. As the structural similarity decreases half of the compound libraries still have an F1 score of at least 0.5 by the top 10 (Supp Fig. 18-19), except for compound libraries within J&J ELN with a Tanimoto similarity of 0.2$-$0.4 which show a slightly lower median of 0.41.

Retrosynthesis is always in the context of feasible chemistry, which goes beyond the similarity of individual compounds within a retrosynthetic route. By assigning reaction types using NameRxn [36] to the proposed reactions, we can assess whether the proposed routes are the same reaction types as the experimentally validated route, despite not going through the same intermediates or building blocks. In this case, reaction name is the most specific naming, with reaction class being one rank above reaction name, where multiple reaction names can fall into a singular reaction class [40]. We find that reaction name accuracy rises to 31.6% and 27.6% for J&J ELN and USPTO at top 5, seeing an increase of over 10% in accuracy compared to the retrosynthetic accuracy in the case of J&J ELN. This further increases slightly when analyzing reaction class, a more granular naming of the reaction type, to 34.4% and 31.4% for J&J ELN and USPTO. Interestingly, we find a minor improvement between reaction name and class. Figure 7 shows two routes that do not have retrosynthetic accuracy yet if we explore the reaction names and classes, we find that 7A proposes the same named reactions as the experimentally validated route, and despite 7B having an F1-score of 0.85 we find that there is an exact match in the reaction classes used. Though it could be assumed that an underlying issue is the proposal of a slightly different reactant, producing the same reaction class but a different reaction name and so different reactant, the difference between retrosynthetic accuracy, reaction name accuracy, and reaction class accuracy shows that there is a more considerable difference in the exact retrosynthetic reactions than the leaving groups of the proposed reactants.

In summary, we present an open-source multi-step synthesis planning framework, which allows the concurrent synthesis of multiple target molecules from compound libraries. Furthermore, we provide the processing and data for a novel analysis and benchmark of the USPTO dataset, focusing on an approach used by medicinal chemists of convergent routes. In particular, this work focuses solely on the output of the single-step model, skewing the search towards convergency. Additionally, other advances in the field such as MCTS can be used to explore the possibility of developing further scoring that identifies compounds that are likely to be common intermediates and useful for convergent synthesis.

## Conclusion

Convergent routes, producing the synthesis of multiple target molecules from a shared synthetic path, are a central effort to accelerate SAR learnings in drug discovery. Here, we introduce a multi-step synthesis planning approach to develop convergent synthesis routes, which can search multiple products and intermediates simultaneously. Convergent synthesis planning can enhance the overall efficiency and practical applicability of retrosynthetic planning, potentially reducing the time and cost of synthesis across compound libraries, while avoiding the combinatorial explosion of combining multiple individual routes. We evaluate the multi-step synthesis planning approach using a novel dataset of convergent routes extracted from industry-relevant and publicly available datasets, showing that over 70% of reactions are found in convergent routes. Using the convergent route approach, we identify a convergent route for over 80% of the test routes and produce a synthesis route for over 97% of compounds. Moreover, the approach shows that the proposed routes are similar to the experimentally validated routes in over a third of the compound libraries, as shown by using a combined F1 score to evaluate retrosynthetic routes.

## Supplementary Information

Supplementary material 1.

## Data Availability

USPTO datasets, including reaction data, building block sets, and convergent routes, along with the code, are available at github.com/aidd-msca/convergent_routes.

## Code availability

All code is available at https://github.com/aidd-msca/convergent_routes.

## References

[1] Corey EJ (1991) The Logic of Chemical Synthesis. John Wiley & Sons Ltd, Hoboken

[2] Irwin R, Dimitriadis S, He J, Bjerrum EJ (2022) Chemformer: A pre-trained transformer for computational chemistry. Machine Learn Sci Technol 3:015022

[3] Tetko IV, Karpov P, Van Deursen R, Godin G (2020) State-of-the-art augmented NLP transformer models for direct and single-step retrosynthesis. Nat Commun 11:5575 33149154 10.1038/s41467-020-19266-y PMC7643129

[4] Chen S, Jung Y (2021) Deep Retrosynthetic Reaction Prediction using Local Reactivity and Global Attention. JACS Au 1:1612–1620 34723264 10.1021/jacsau.1c00246 PMC8549044

[5] Zhong Z et al (2022) Root-aligned SMILES: A tight representation for chemical reaction prediction. Chem Sci 13:9023–9034 36091202 10.1039/d2sc02763a PMC9365080

[6] Zhong Z et al (2023). Recent advances in artificial intelligence for retrosynthesis. arxiv:2301.05864

[7] Heifets A, Jurisica I (2012) Construction of New Medicines via Game Proof Search. Proc AAAI Conf Artif Intell 26:1564–1570

[8] Kishimoto A, Buesser B, Chen B, Botea A (2019) Depth-first proof-number search with heuristic edge cost and application to chemical synthesis planning. Adv Neural Inf Process Syst 32:1

[9] Segler MHS, Preuss M, Waller MP (2018) Planning chemical syntheses with deep neural networks and symbolic AI. Nature 555:604–610 29595767 10.1038/nature25978

[10] Lin K, Xu Y, Pei J, Lai L (2020) Automatic retrosynthetic route planning using template-free models. Chem Sci 11:3355–3364 34122843 10.1039/c9sc03666k PMC8152431

[11] Ishida S, Terayama K, Kojima R, Takasu K, Okuno Y (2022) AI-Driven Synthetic Route Design Incorporated with Retrosynthesis Knowledge. J Chem Inf Model 62:1357–1367 35258953 10.1021/acs.jcim.1c01074 PMC8965881

[12] Saigiridharan L et al (2024) AiZynthFinder 4.0: Developments based on learnings from 3 years of industrial application. J Cheminf 16:57 10.1186/s13321-024-00860-x PMC11112899 38778382

[13] Chen B, Li C, Dai H, Song L (2020). Retro*: Learning Retrosynthetic Planning with Neural Guided A* Search

[14] Xie S et al (2022). Retrograph: Retrosynthetic planning with graph search. In Proceedings of the 28th ACM SIGKDD Conference on Knowledge Discovery and Data Mining (pp. 2120–2129)

[15] Kim J, Ahn S, Lee H, Shin J (2021) Self-Improved Retrosynthetic Planning. In International Conference on Machine Learning (pp. 5486–5495)

[16] Schreck JS, Coley CW, Bishop KJ (2019) Learning retrosynthetic planning through simulated experience. ACS Central Sci 5:970–981 10.1021/acscentsci.9b00055 PMC6598174 31263756

[17] Dandapani S, Rosse G, Southall N, Salvino JM, Thomas CJ (2012) Selecting, Acquiring, and Using Small Molecule Libraries for High-Throughput Screening. Curr Protoc Chem Biol 4:177–191 26705509 10.1002/9780470559277.ch110252 PMC4687755

[18] Brown DG, Boström J (2018) Where Do Recent Small Molecule Clinical Development Candidates Come From? J Med Chem 61:9442–9468 29920198 10.1021/acs.jmedchem.8b00675

[19] Seneci P (2018) in Chapter 4 - Step IIIb: The Drug-Like Chemical Diversity Pool: Diverse and Targeted Compound Collections. In: Seneci P (ed) Chemical Sciences in Early Drug Discovery. Elsevier, Oxford, pp 115–177

[20] Fromer JC, Coley CW (2024) An algorithmic framework for synthetic cost-aware decision making in molecular design. Nat Comput Sci 4:440–450 38886590 10.1038/s43588-024-00639-y

[21] Coley CW et al (2019) A robotic platform for flow synthesis of organic compounds informed by AI planning. Science. 2019; 365: 6453 10.1126/science.aax1566 31395756

[22] Pasquini M, Stenta M (2023) LinChemIn: SynGraph–a data model and a toolkit to analyze and compare synthetic routes. J Cheminf 15:41 10.1186/s13321-023-00714-y PMC10067316 37005691

[23] Gao H, Pauphilet J, Struble TJ, Coley CW, Jensen KF (2021) Direct Optimization across Computer-Generated Reaction Networks Balances Materials Use and Feasibility of Synthesis Plans for Molecule Libraries. J Chem Inf Model 61:493–504 33331158 10.1021/acs.jcim.0c01032

[24] Gao H et al (2020) Combining retrosynthesis and mixed-integer optimization for minimizing the chemical inventory needed to realize a WHO essential medicines list. React Chem Eng 5:367–376

[25] Molga K, Dittwald P, Grzybowski AB (2019) Computational design of syntheses leading to compound libraries or isotopically labelled targets. Chem Sci 10:9219–9232 32055308 10.1039/c9sc02678a PMC6979321

[26] Szymkuć S et al (2016) Computer-Assisted Synthetic Planning: The End of the Beginning. Angewandte Chemie Int Edition 55:5904–5937 10.1002/anie.201506101 27062365

[27] Lowe DM (2012). Extraction of Chemical Structures and Reactions from the Literature. Thesis

[28] Genheden S, Bjerrum E (2022) PaRoutes: Towards a framework for benchmarking retrosynthesis route predictions. Digital Discov 1:527–539

[29] Mo Y et al (2021) Evaluating and clustering retrosynthesis pathways with learned strategy. Chem Sci 12:1469–1478 10.1039/d0sc05078d PMC8179211 34163910

[30] Tripp A, Maziarz K, Lewis S, Liu G, Segler M (2022). Re-Evaluating Chemical Synthesis Planning Algorithms

[31] Hassen AK et al (2022). Mind the Retrosynthesis Gap: Bridging the divide between Single-step and Multi-step Retrosynthesis Prediction . arxiv:2212.11809

[32] Neves P et al (2023) Global reactivity models are impactful in industrial synthesis applications. J Cheminf 15:20 10.1186/s13321-023-00685-0 PMC9921076 36774523

[33] RDKit. https://www.rdkit.org/

[34] Nugmanov R, Dyubankova N, Gedich A, Wegner JK (2022) Bidirectional Graphormer for Reactivity Understanding: Neural Network Trained to Reaction Atom-to-Atom Mapping Task. J Chem Inf Model 62:3307–3315 35792579 10.1021/acs.jcim.2c00344

[35] Torren-Peraire P et al (2024) Models Matter: The impact of single-step retrosynthesis on synthesis planning. Digit Discov 3:558–572

[36] NameRxn. NextMove Software

[37] Schneider N, Lowe DM, Sayle RA, Tarselli MA, Landrum GA (2016) Big Data from Pharmaceutical Patents: A Computational Analysis of Medicinal Chemists’ Bread and Butter. J Med Chem 59:4385–4402 27028220 10.1021/acs.jmedchem.6b00153

[38] eMolecules, Inc. eMolecules Chemical Building Blocks. https://www.emolecules.com/products/building-blocks (2024)

[39] Schwaller P et al (2020) Predicting retrosynthetic pathways using transformer-based models and a hyper-graph exploration strategy. Chem Sci 11:3316–3325 34122839 10.1039/c9sc05704h PMC8152799

[40] Lagersted I, Mayfiel J, Sayl R (2021). NameRxn More than just a Reaction Classifier

