# Supplementary material for: Improving route development using convergent retrosynthesis planning
Source: J Cheminform. 2025 Feb 27;17:26. doi: 10.1186/s13321-025-00953-1 (PMC11869726; doi:10.1186/s13321-025-00953-1)
Supplement: Supplementary file 1 — Supplementary material 1. [file 13321_2025_953_MOESM1_ESM.pdf]

Appendix A Extended Data

$$\text{Accuracy} = \frac{1}{n}\sum_{i=1}^{n} f(E_e, E_p)$$
$$f(E_e, E_p) = \begin{cases} 1, & \text{if } E_\text{p} = E_\text{e} \\ 0, & \text{otherwise} \end{cases} \tag{A1}$$

$$F_1(E) = \frac{2|E_p \cap E_e|}{2|E_p \cap E_e| + |E_p \setminus E_e| + |E_e \setminus E_p|} \tag{A2}$$

$$V_p = V_p \setminus V_{tm}$$
$$V_e = V_e \setminus V_{tm}$$
$$F_1(V) = \frac{2|V_p \cap V_e|}{2|V_p \cap V_e| + |V_p \setminus V_e| + |V_e \setminus V_p|} \tag{A3}$$

$$F_1 = \frac{F_1(E) + F_1(V)}{2} \tag{A4}$$

Algorithm 1 Convergent Search Algorithm

```
1:  convergent_search = add_molecule_nodes(TARGET_MOLECULES)
    ▷ Initialize the graph with all target molecules
2:  selected_nodes = get_end_nodes(convergent_search)
    ▷ Select all target molecules as selected nodes
3:  for _ in MAXIMUM_ITERATIONS do
4:      for node in selected_nodes do
5:          reactants, probability = SINGLE_STEP_MODEL(node)
6:          reaction_node = create_reaction_node(node, probability)
7:          molecule_nodes = create_molecule_node(reaction_node, reactants)
8:          edges = create_edges(node, reaction_node, molecule_nodes)
9:          convergent_search.update(reaction_node, molecule_nodes, edges)
10:     end for
11:     end_nodes = get_end_nodes(NODES)
        ▷ Gather all nodes that are:
        ▷ - unexplored
        ▷ - within max route length
        ▷ - not in building block set
12:     end_nodes = score_end_nodes(end_nodes)
        ▷ Score end nodes based on Eq. 5
13:     selected_nodes = K_highest_ranked_nodes(end_nodes, K)
        ▷ Select highest ranked nodes
14: end for
```

Fig. A1 Pseudocode for convergent search development algorithm. This code iteratively expands the convergent search through selected node expansion. Lines 4-10 are carried out in batches but are here shown as a for loop for ease of reading, additionally the stop criterion have not been included and only maximum number of iterations is considered.

Algorithm 2 Score End Nodes

```
procedure SCORE_END_NODES(end_nodes)
    for node in end_nodes do
        target_paths = shortest_paths(node, TARGET_MOLECULES)
        ▷ Compute shortest linear paths
        scores = []
        ▷ Initialize list for scores
        for path in target_paths do
            probabilities = product(path.reaction_nodes)
            ▷ Calculate path probabilities
            S = max(probabilities)
            ▷ Select highest probability path
            scores.append(S)
            ▷ Append path score
        end for
        score = sum(scores)/len(scores)
        ▷ Compute average score
        node.score = score
        ▷ Assign score to node
    end for
    return end_nodes
    ▷ Return updated nodes
end procedure
```

Fig. A2 Pseudocode for selected end node scoring. This code scores the end nodes based on the single-step model scores, averaging across target molecules, as defined in Eq. 5.

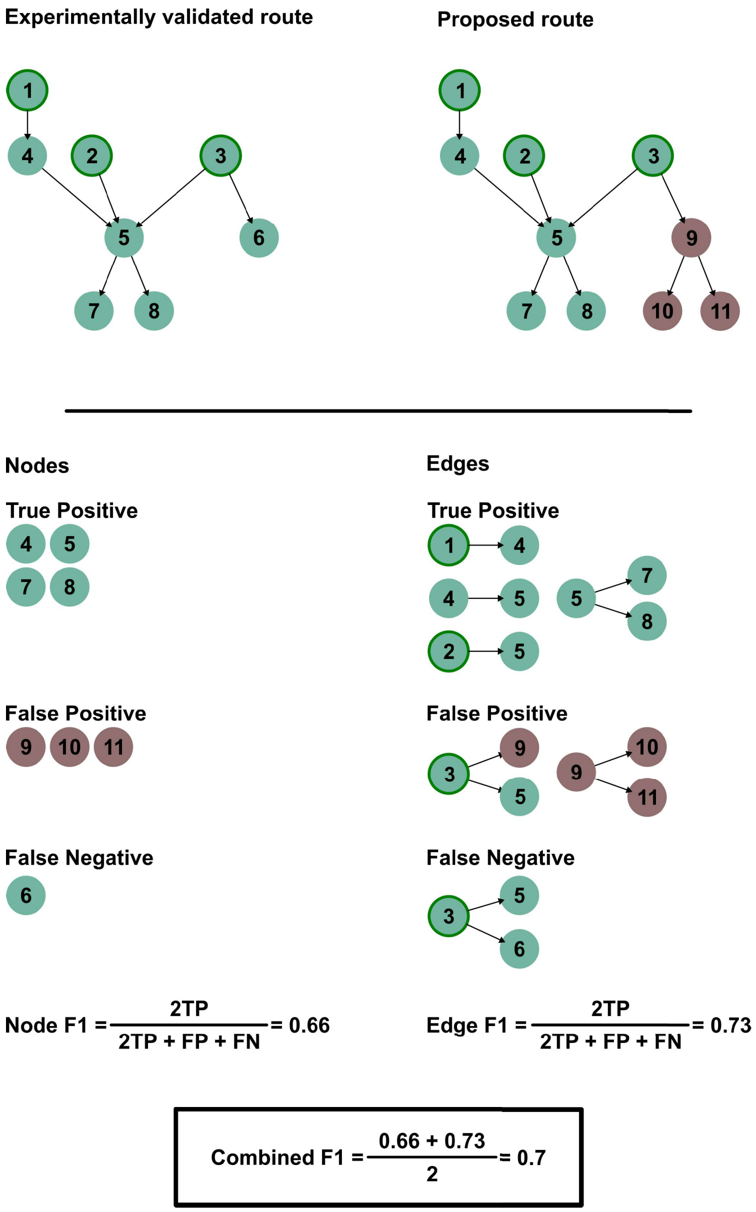

Fig. A3 Visual representation of F1 score calculation. Green nodes are present in the experimentally validated route, brown nodes preset in the proposed route deviate from the experimentally validated route, nodes with dark green border are target molecules.

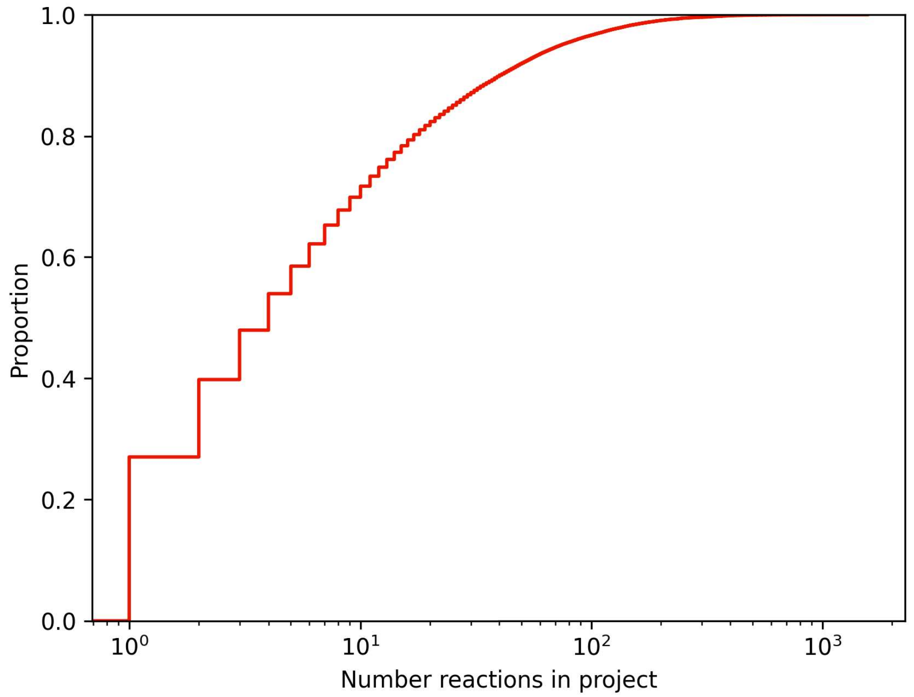

Fig. A4 Proportion of USPTO projects with the associated number of reactions.

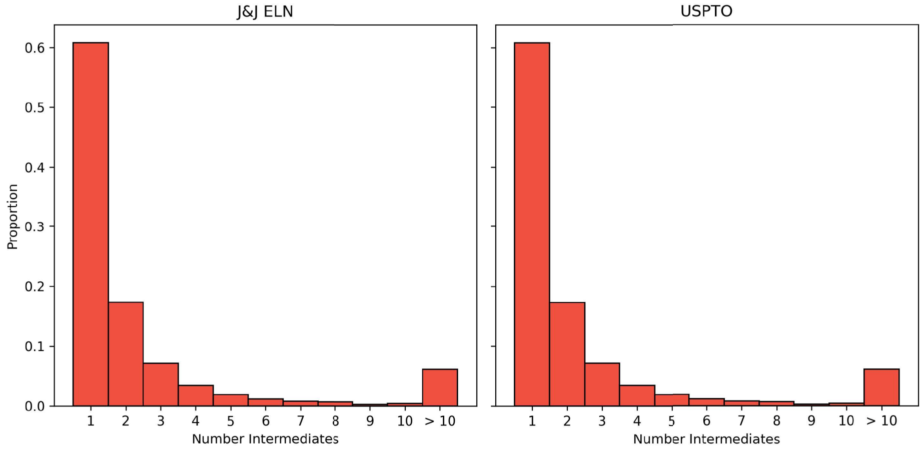

Fig. A5 Number of intermediate compounds per convergent route as a proportion of all convergent routes per dataset.

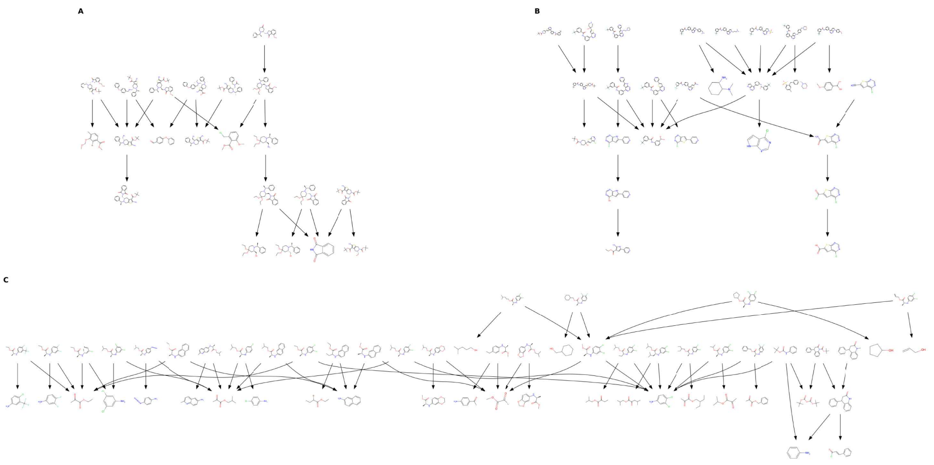

Fig. A6 Synthesis routes of routes exemplified in Fig. 2.

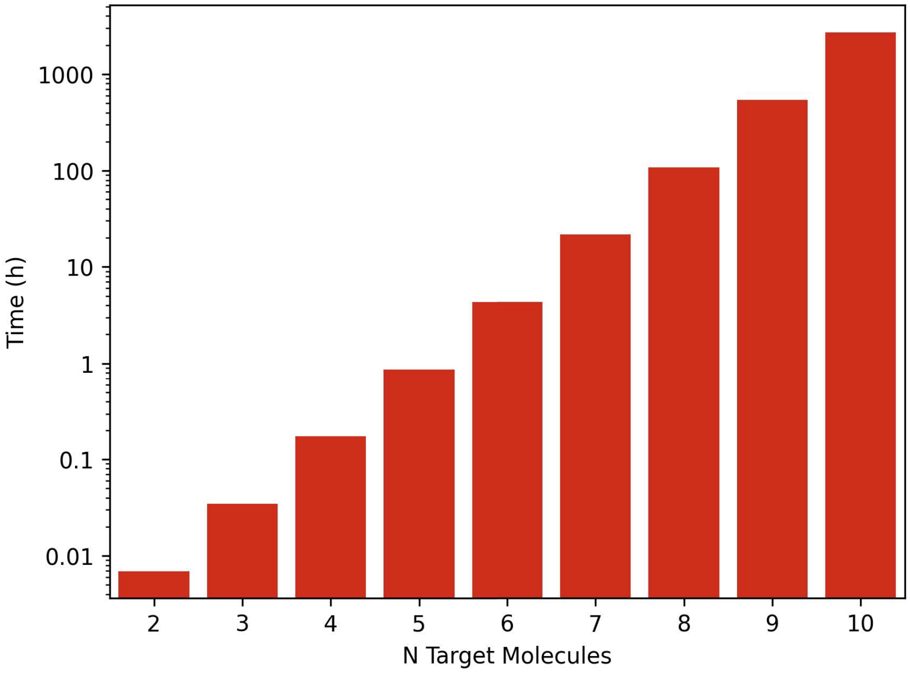

Fig. A7 Combinatorial effect of increasing number of target molecules, assuming one second per combination on top 5 routes per target molecule.

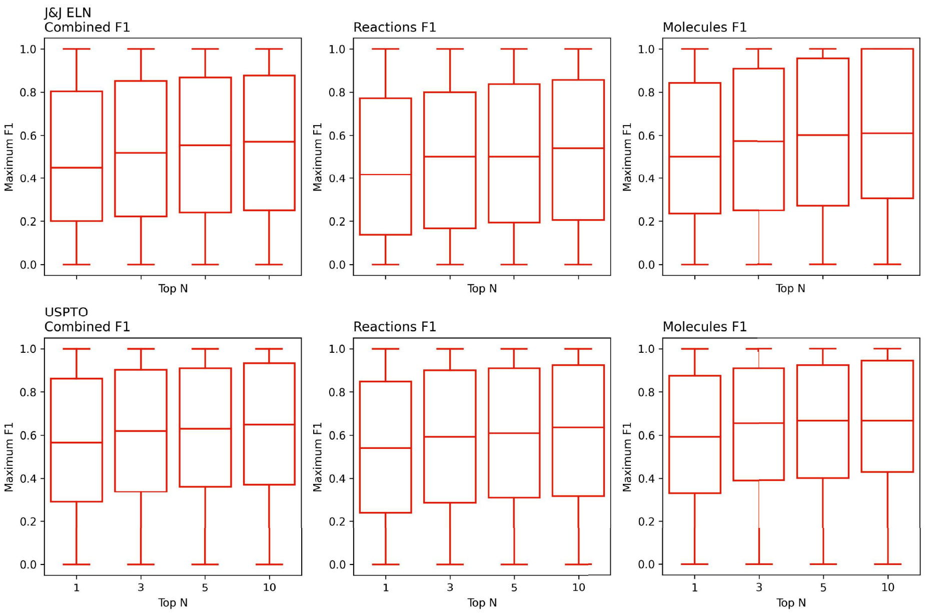

Fig. A8 F1 score based on combined (as shown in main text), molecule (node) and reaction (edge) calculation for J&J ELN and USPTO routes.

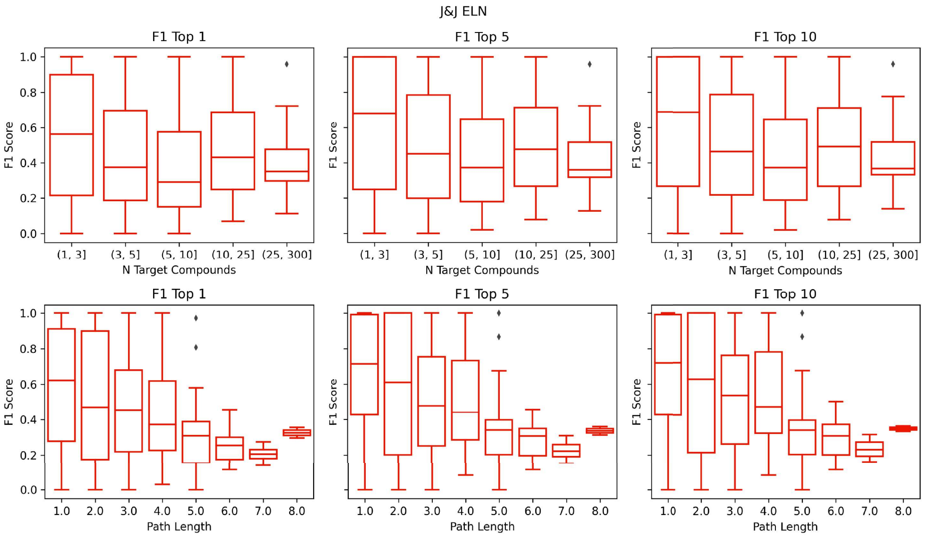

Fig. A9 F1 score of J&J ELN routes in relation to the number of target compounds (top panel) and maximum path length (bottom panel) at top 1, top 5, and top 10.

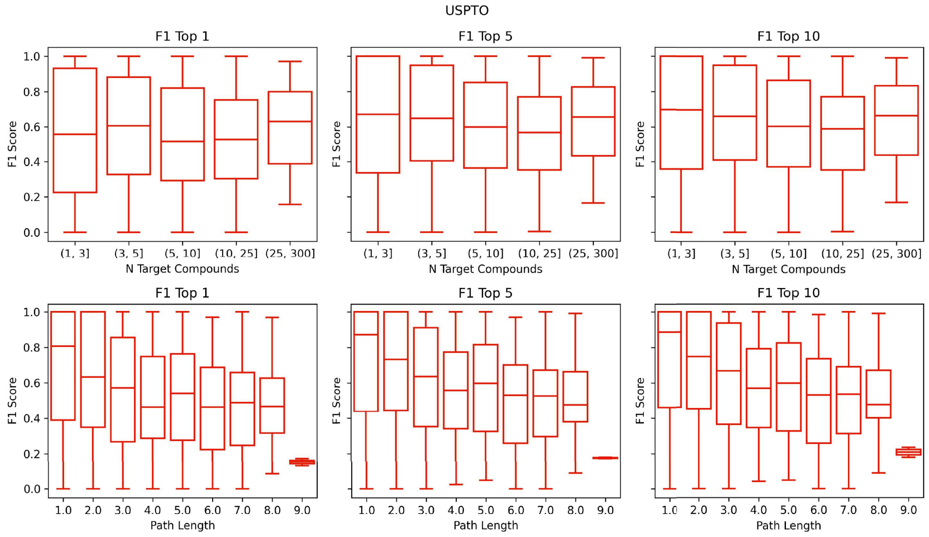

Fig. A10 F1 score of USPTO routes in relation to the number of target compounds (top panel) and maximum path length (bottom panel) at top 1, top 5, and top 10.

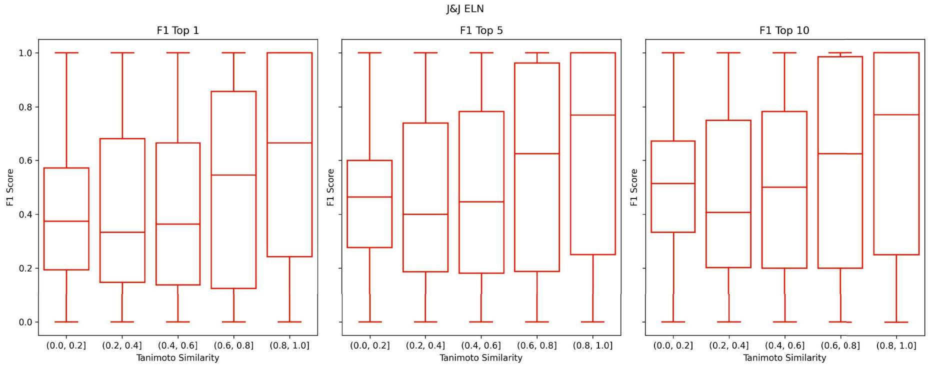

Fig. A11 F1 score of J&J ELN routes in relation to Tanimoto similarity of target compounds at top 1, top 5, and top 10.

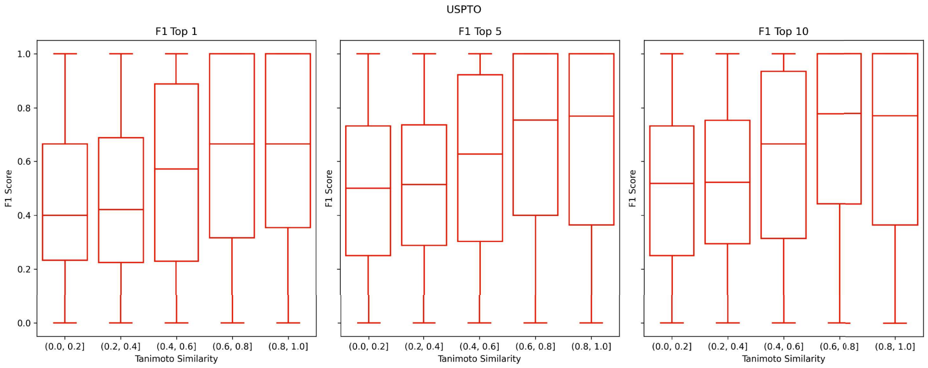

Fig. A12 F1 score of USPTO routes in relation to Tanimoto similarity of target compounds at top 1, top 5, and top 10.

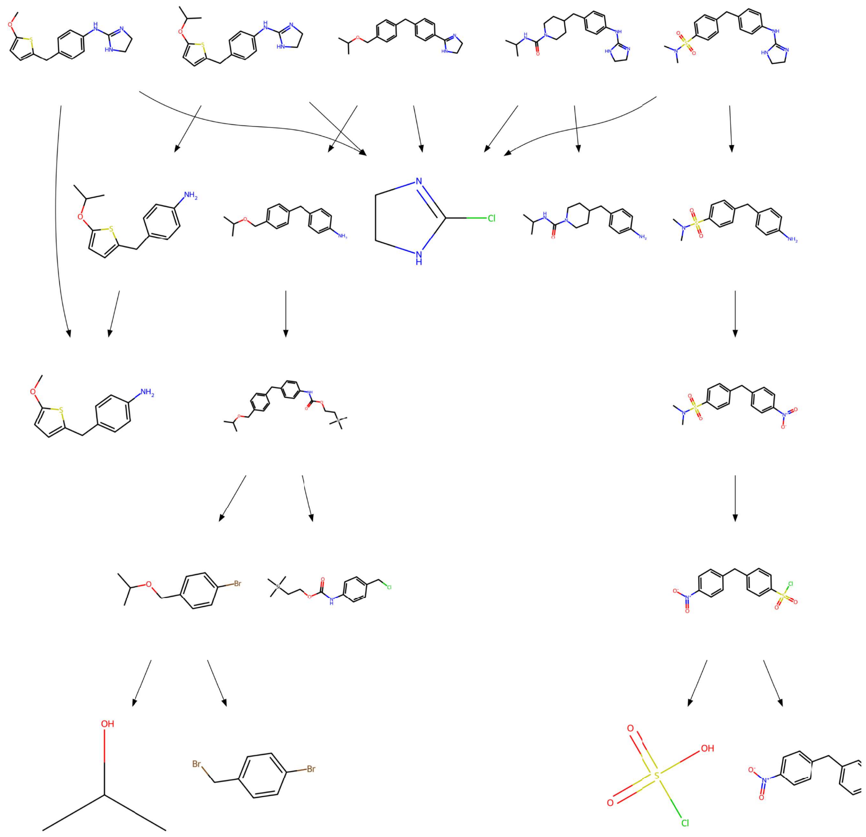

Fig. A13 Full-scale figure of experimentally validated route shown in Fig. 6 panel A.

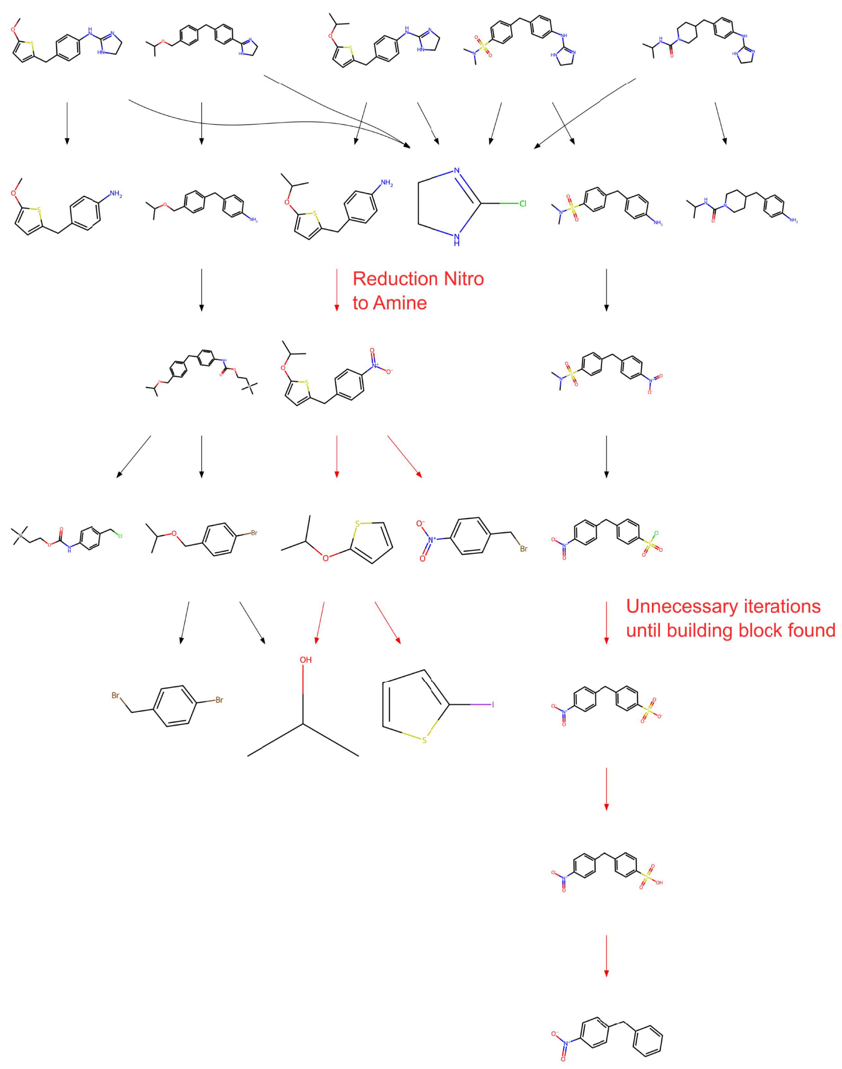

Fig. A14 Full-scale figure of proposed route shown in Fig. 6 panel B.

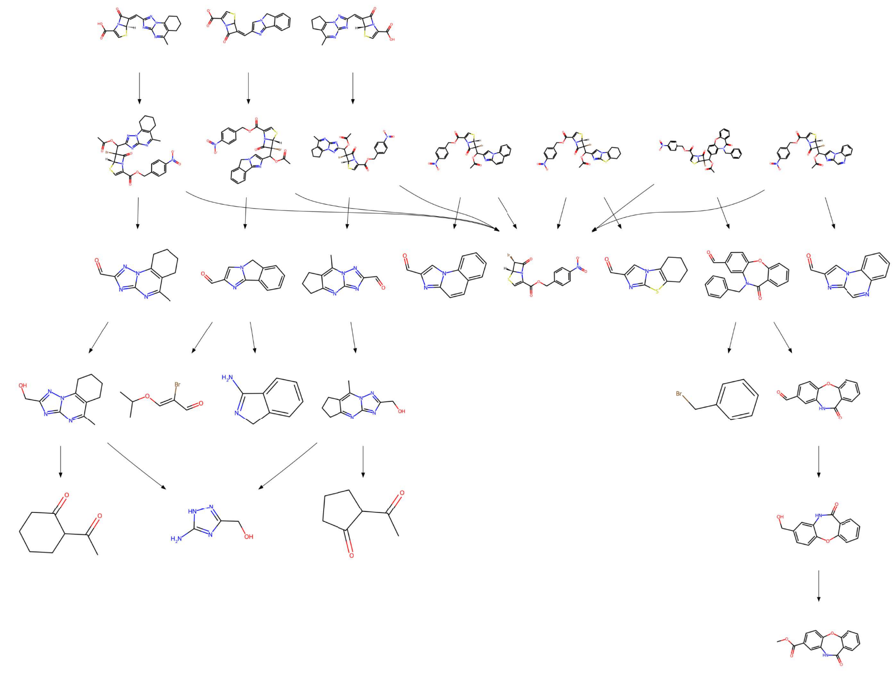

Fig. A15 Full-scale figure of experimentally validated route shown in Fig. 6 panel B.

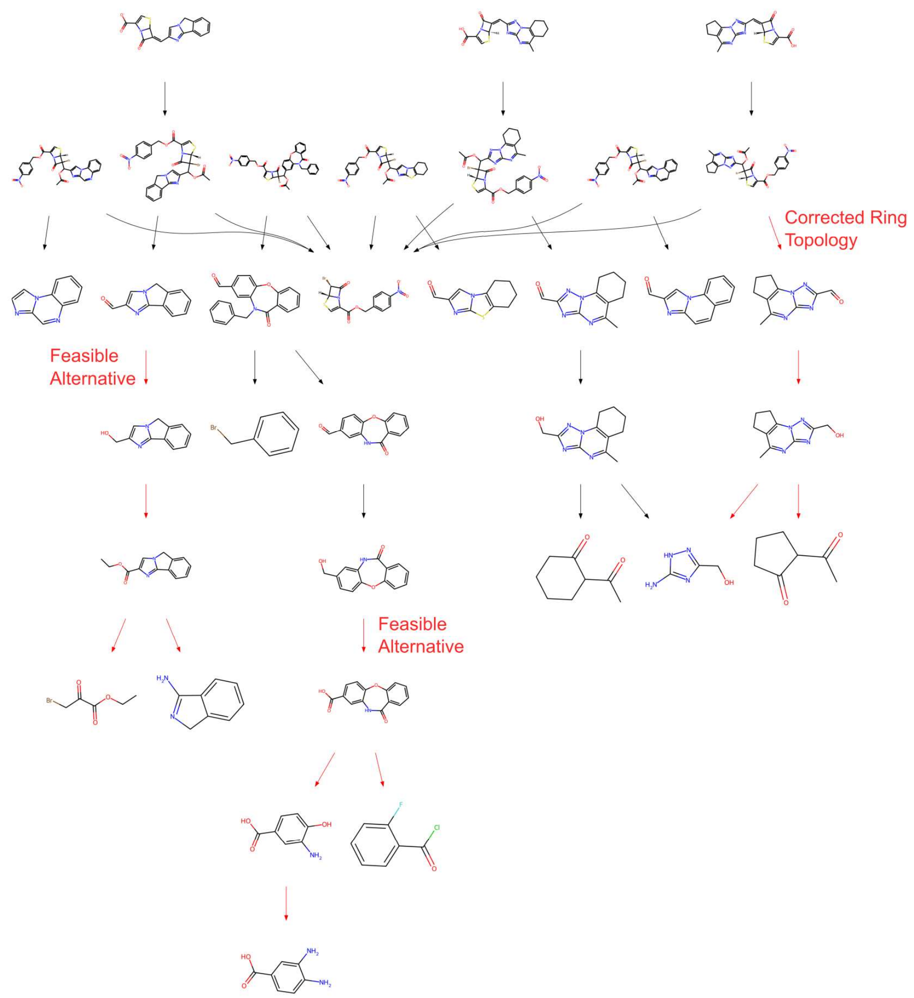

Fig. A16 Full-scale figure of proposed route shown in Fig. 6 panel B.

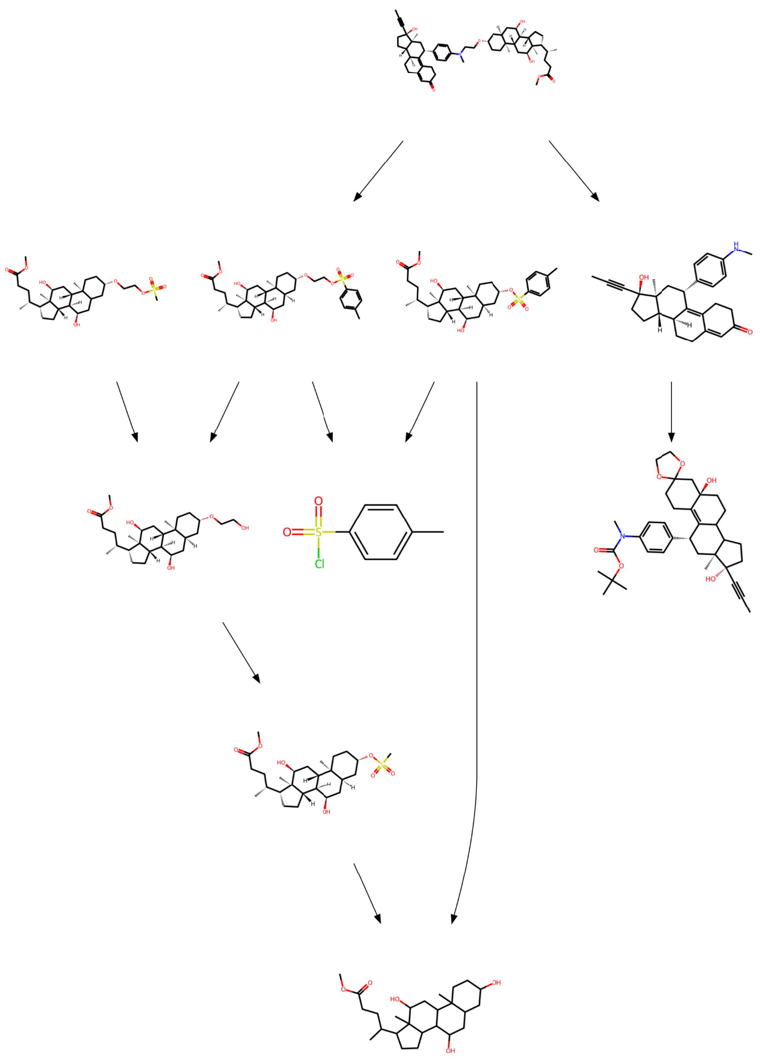

Fig. A17 Full-scale figure of experimentally validated route shown in Fig. 7 panel A.

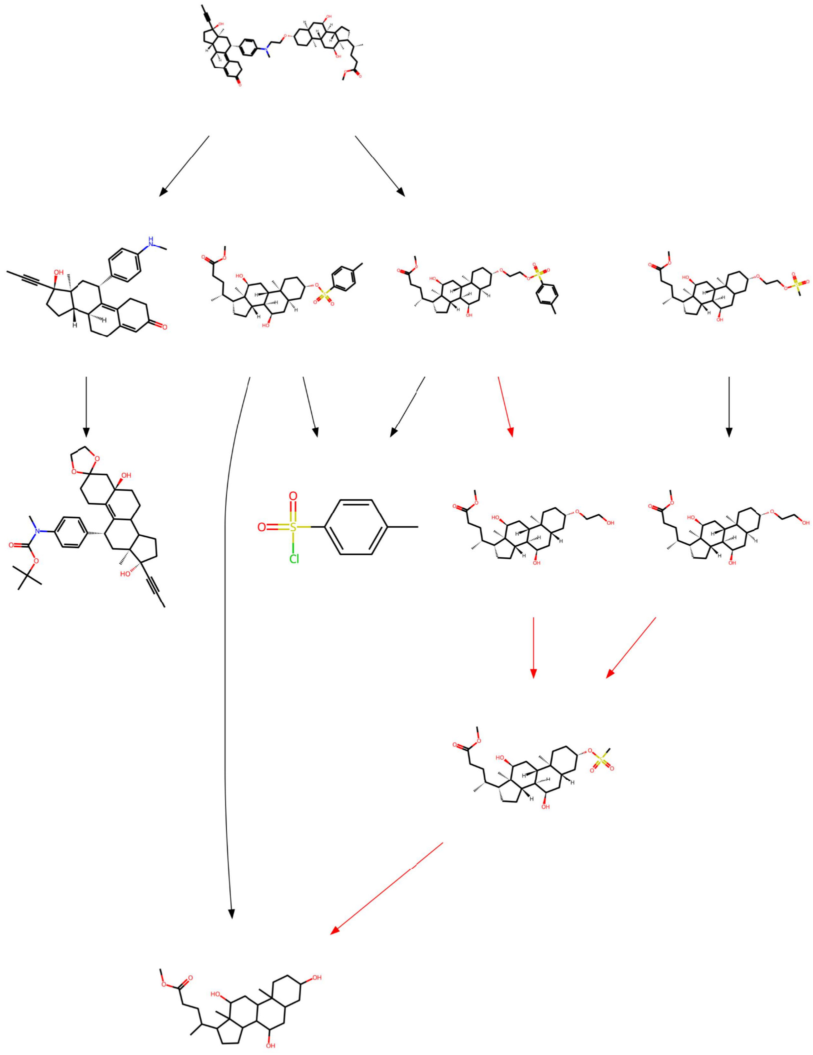

Fig. A18 Full-scale figure of proposed route shown in Fig. 7 panel B.

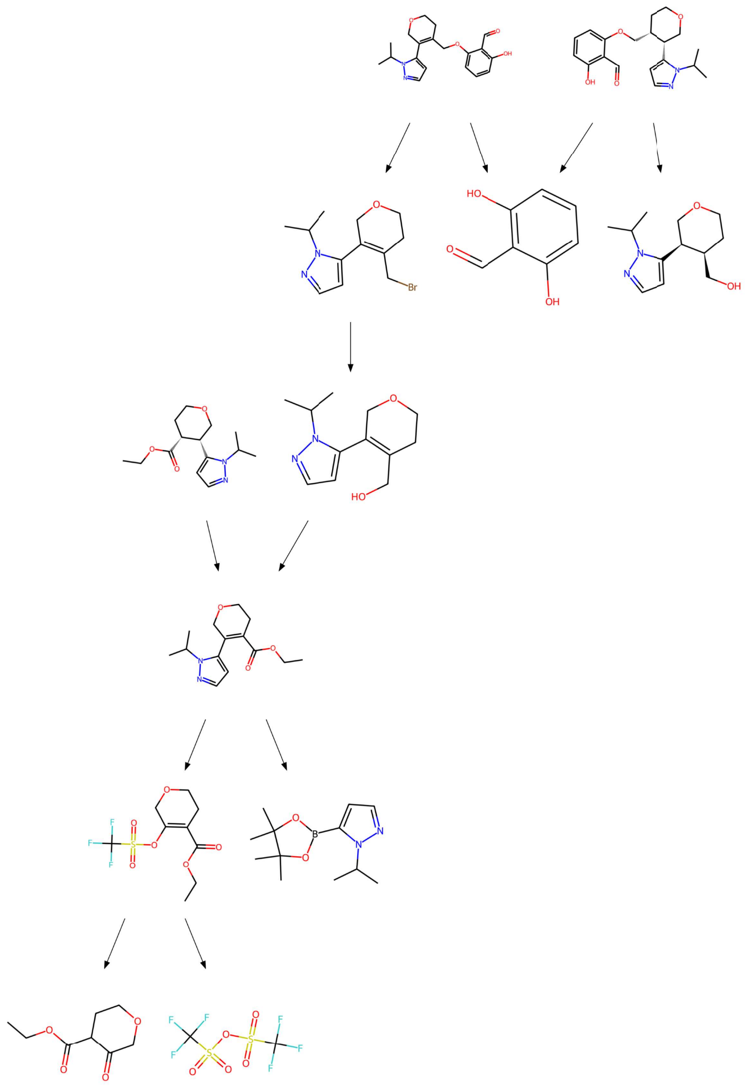

Fig. A19 Full-scale figure of experimentally validated route shown in Fig. 7 panel B.

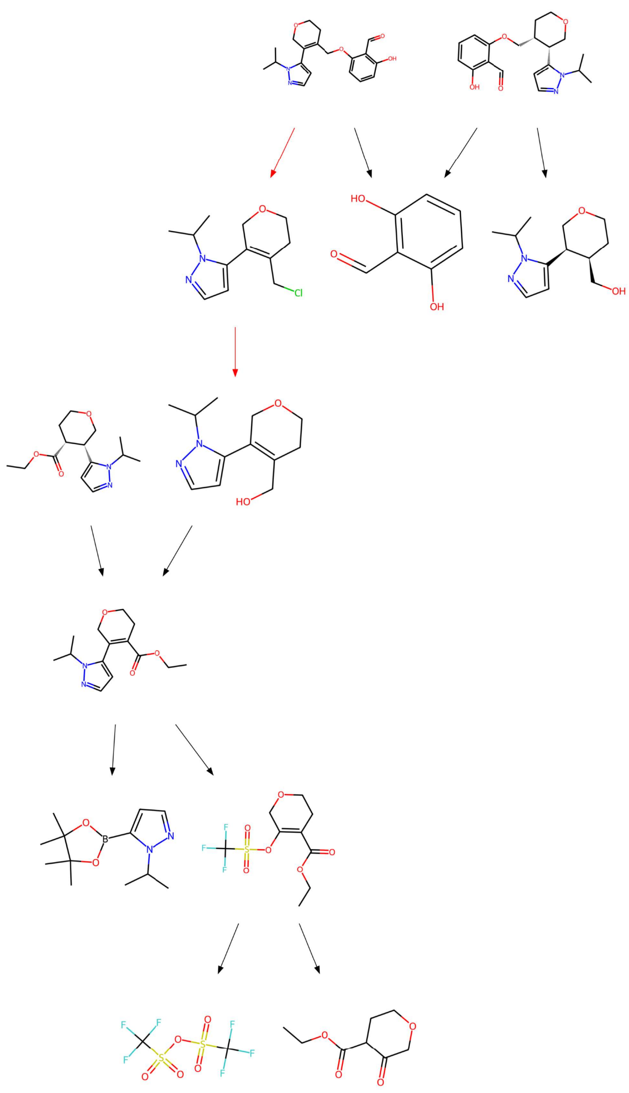

Fig. A20 Full-scale figure of proposed route shown in Fig. 7 panel B.
